# Reusable nano-catalyzed green protocols for the synthesis of quinoxalines: an overview

**DOI:** 10.1039/d3ra03646d

**Published:** 2023-07-07

**Authors:** Rangappa S. Keri, Dinesh Reddy, Srinivasa Budagumpi, Vinayak Adimule

**Affiliations:** a Centre for Nano and Material Sciences, Jain (Deemed-to-be University) Jain Global Campus, Kanakapura Bangalore Karnataka 562112 India keriphd@gmail.com sk.rangappa@jainuniversity.ac.in +918027577199 +919620667075; b Angadi Institute of Technology and Management (AITM) Savagaon Road Belagavi-5800321 Karnataka India

## Abstract

Heterocyclic compounds are very widely distributed in nature and are essential for life activities. They play a vital role in the metabolism of all living cells, for example, vitamins and co-enzyme precursors thiamine, riboflavin *etc.* Quinoxalines are a class of N-heterocycles that are present in a variety of natural and synthetic compounds. The distinct pharmacological activities of quinoxalines have attracted medicinal chemists considerably over the past few decades. Quinoxaline-based compounds possess extensive potential applications as medicinal drugs, presently; more than fifteen drugs are available for the treatment of different diseases. Diverse synthetic protocols have been developed *via* a one-pot approach using efficient catalysts, reagents, and nano-composites/nanocatalysts *etc.* But the use of homogeneous and transition metal-based catalysts suffers some demerits such as low atom economy, recovery of catalysts, harsh reaction conditions, extended reaction period, expensive catalysts, the formation of by-products, and unsatisfactory yield of products as well as toxic solvents. These drawbacks have shifted the attention of chemists/researchers to develop green and efficient protocols for synthesizing quinoxaline derivatives. In this context, many efficient methods have been developed for the synthesis of quinoxalines using nanocatalysts or nanostructures. In this review, we have summarized the recent progress (till 2023) in the nano-catalyzed synthesis of quinoxalines using condensation of *o*-phenylenediamine with diketone/other reagents with plausible mechanistic details. With this review, we hope that some more efficient ways of synthesizing quinoxalines can be developed by synthetic chemists.

## Abstract 2

Heterocyclic compounds are very widely distributed in nature and are essential for life activities.

## Graphical abstract

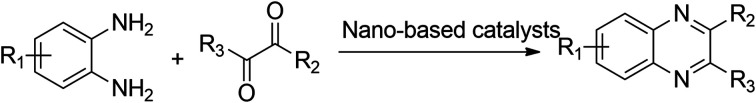

## 1 Introduction

**Fig. 1:**
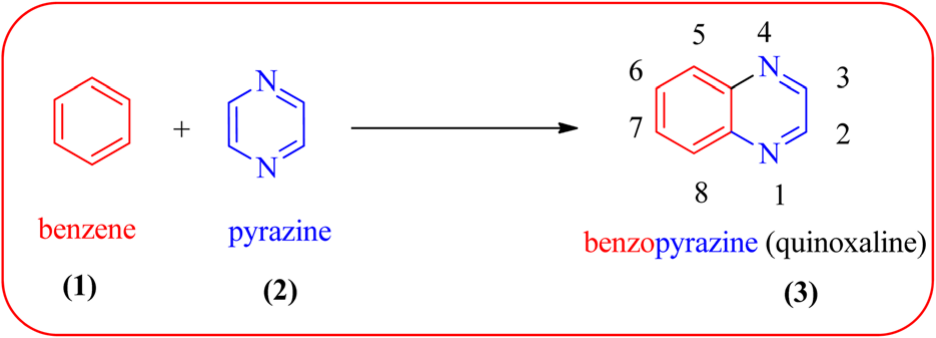
Numbering system in quinoxalines.

Heterocyclic compounds are one of the hot/vast research topics for organic/medicinal chemists. Heterocycles play an important role in the field of drug discovery, biochemistry, materials chemistry, and other areas of science.^1^ The majority of heterocyclic compounds found in nature contain nitrogen, making them the richest class of compounds compared to those containing oxygen or sulfur.^2^ Among nitrogen-containing heterocycles, quinoxalines (3) are considered a privileged structure in medicinal chemistry because of their broad pharmacological/biological activity. Quinoxalines, also known as benzopyrazines (a benzodiazine family), have a bicyclic ring system made up of a benzene ring (1) fused with a pyrazine ring (2) (Fig. 1).

The compounds with a quinoxaline motif display an array of biological and pharmaceutical activities, such as antimicrobial,^3^ antitumor,^4^ antituberculosis,^5^ anti-HIV,^6^ antiproliferative,^7^ anticancer,^8^ antileishmanial,^9^ anti-inflammatory,^10,11^ antiviral,^12^ anti-protozoal,^13^ anthelmintic,^14^ antifungal,^15^ analgesic,^16^ antimalarial,^17^ antiepileptic,^18^ anticonvulsant,^19^ antiamoebic,^20^ anti-HCV,^21^ kinase inhibitors,^22^ antidepressant^23^ and *etc.* Quinoxaline cores also find prominent uses in agrochemicals as insecticides,^24^ pesticides,^25^ herbicides.^26^ Other than biological/pharmacological applications, these structural moieties have also found applications in phosphorescence light emitting diodes, solar cells,^27^ dyes,^28^ organic semiconductors,^29^ chemically controllable switches,^30^ efficient electron luminescent material,^31^ cavitands,^32^ building blocks for the synthesis of anion receptor,^33^ dehydoannulenes,^34^ DNA cleaving agents^35^ and also for the development of macrocyclic molecular receptors.^36,37^

**Fig. 2:**
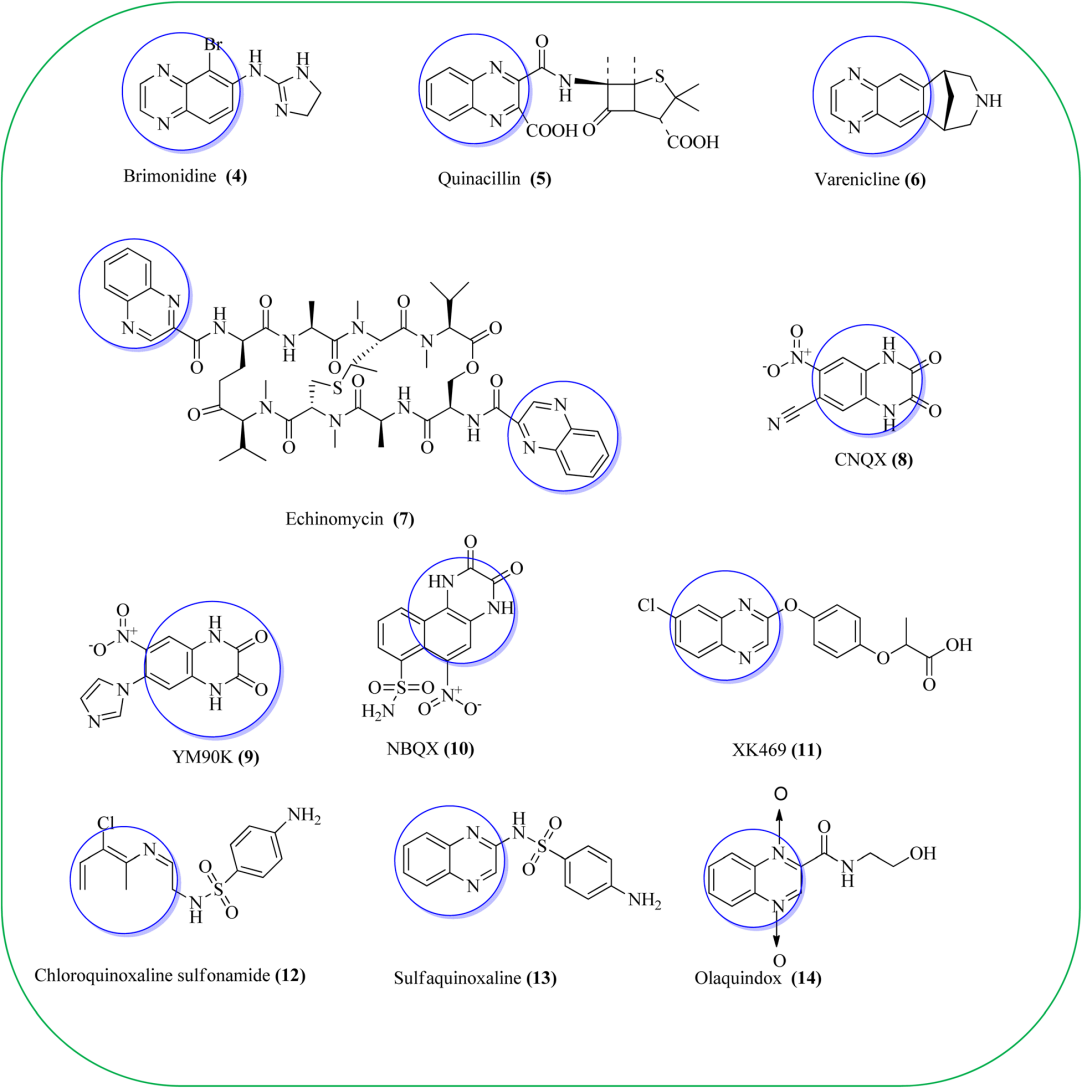
Marketed drugs having quinoxaline ring (highlighted).

Some of the quinoxaline scaffolds are the parts of commercially marketed drugs to quote a few: brimonidine (4, used to treat open-angle glaucoma, ocular hypertension, and rosacea),^38^ quinacillin (5, penicillin antibiotic which can reversibly deactivate beta-lactamase enzymes),^39^ varenicline (6, agonist at nicotinic acetylcholine receptors used as an aid in smoking cessation),^40^ echinomycin or levomycin/quinomycin A (7, peptide antibiotic),^41^ 6-cyano-7-nitroquinoxaline-2,3-dione (8, CNQX, AMPA/kainate receptor antagonist),^42^ YM90K (9, AMPA receptor antagonis),^43^ NBQX (10, AMPA receptor antagonist),^44^ XK469 (11, anti-tumor agent, effects by topoisomerase IIB inhibition),^45^ chloroquinoxaline sulfonamide (12, CQS, NSC 339004, antineoplastic),^46^ sulfaquinoxaline (13, veterinary medicine, to treat coccidiosis),^47^ and olaquindox (14, growth stimulant) (Fig. 2).^48^

**Scheme 1:**
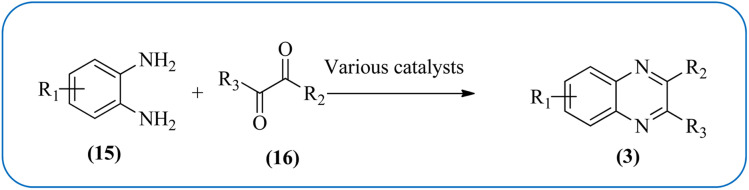
General scheme for synthesis of quinoxalines by the condensation of 1,2-dicarbonyl and aryl 1,2-diamines.

Therefore, the synthesis of quinoxalines have received increasing attention from synthetic organic chemists and biologists. A variety of methods for the synthesis of quinoxaline derivatives have been developed/reported, for instance, the reaction between aryl-1,2-diamines and diethyl bromomalonate,^49^ reductive cyclizations of 1,2-dicarbonyl compounds with 2-nitroanilines,^50^ the reaction of aromatic 1,2-diamines with α-halo ketones,^51^ the coupling of aryl 1,2-diamines with α-diazo ketones,^52^ reactions between OPD and α-hydroxy ketones,^53^ oxidative cyclizations of heteroannulation of nitroketene *N*,*S*-aryliminoacetals with POCl_3_,^54^ the reaction of 1,2-diamines and α-keto oximes,^55^ intramolecular cyclizations of dialdimines,^56^ oxidative coupling of ene-1,2-diamines and epoxides,^57^ and alkynes with 1,2-diamines *via* key oxidation.^58,59^ The most simple and straightforward route for the synthesis of quinoxaline derivatives (1) is the direct condensation of 1,2-dicarbonyl compounds (16) and aryl 1,2-diamines (15) (Scheme 1). For this transformation, a variety of catalysts were used *viz.*, sulfamic acid,^60^ oxalic acid,^61^ montmorillonite K-10,^62^ cerium(iv) ammonium nitrate,^63^ Wells–Dawson hetero polyacid,^64^ polyanilinesulfate salt,^65^ gallium(iii) triflate,^66^ ionic liquid 1-*n*-butylimidazolium tetrafluoroborate,^67^ molecular iodine,^68^ bismuth(iii) triflate,^69^ indium chloride,^70^ zirconium tetrakis(dodecyl sulfate),^71^ molybdophosphoric acid,^72^ zeolites,^73^ graphite,^74^ palladium,^75^ copper,^76^ iron exchanged^77^ and, many more. Although, these aforementioned protocols unfold several remarkable features, the intrinsic difficulties arising due to the use of strong acids, costlier reagents, oxidants, elevated temperature, long reaction time, use of toxic solvents, the separation of catalyst from the reaction system, and reusability of homogeneous catalysts, inescapably add to the cost and even render its bleak commercial utility due to presence of these metal residues in the product stream.

To overcome these demerits, nanomaterials/nanocatalysts have emerged as better alternatives for several organic transformations as compared to conventional materials applicable to almost all types of catalytic organic transformations. In the last two decades, nanoparticles/nanocatalysts gained more attention to scientists to do more attractive innovations in diverse fields, including energy conversion and storage, chemical manufacturing, biological applications, semiconductors, photocatalysts, nanobots, and environmental technology. Among nanocatalysts, several forms such as nano-supported catalysts, graphene-based nanocatalysts, nano-mixed metal oxides, magnetic nanocatalysts, and core–shell nanocatalysts have been employed in catalytic applications.^78^ Nanoparticle-supported catalysts/nanostructures can offer important advantages as compared to homogeneous transition metal systems. These include good reusability coupled with high activities, low preparation cost, great selectivity, high stability, excellent activity, and specificities in different chemistries based on their excelling properties (high surface areas, degenerated density of energy states, and plasmon). A few review articles have already been published revealing varied pharmacological/and biological applications of quinoxaline and its derivatives^79–81^ and the application of transition metal for the synthesis of quinoxalines and also nano-catalysts with metal single-atom catalysts.^82,83^ These metal-based catalysts suffers some disadvantages like recovery and low yield. So, this review gives a comprehensive insight into the application of nanocatalysts for the synthesis of various substituted quinoxalines by the condensation of 1,2-dicarbonyl compounds and aryl 1,2-diamines and their plausible reaction mechanisms reported since 2000. The present review has been categorized depending on the types of nanocatalysts (like Fe, Ti, Cu, Co, Mn, Mo, Au, Ag, Zn, Zr, Si, Pd, and Ni-based nanocatalysts) used for the synthesis of quinoxaline. Representative examples of each category used for the synthesis of compounds bearing a quinoxaline core with the plausible mechanism of their synthetic assembly have been documented.

## 2 Synthesis of quinoxaline using nano-based catalyst

### 2.1. Iron-based nanocatalysts for the synthesis of quinoxalines

Iron-based nanoparticles have unique advantages such as high surface area, abundant, nontoxic, readily accessible, and retrievable which offer attractive supports for immobilizing various functional groups under mild conditions. Also, due to the magnetic properties of iron oxide, it provides a simpler work-up procedure in the separation stages with the use of an external magnet over filtration or centrifugation both in preparation and recovery.^84^ Also, Fe NPs used in a wide range of applications like dechlorination, potential magnetic carriers, electrocatalysis, magnetic resonance imaging, remediation of aqueous metal contaminants, chromium removal, and in organic syntheses like imine synthesis, and various heterocyclic motifs.^85,86^ Fe-based nanomaterials were used for many organic transformations or heterocyclic synthesis.^87^ Also, many Fe-based catalysts/materials were used for the synthesis of quinoxalines and are discussed in this section.

**Scheme 2:**
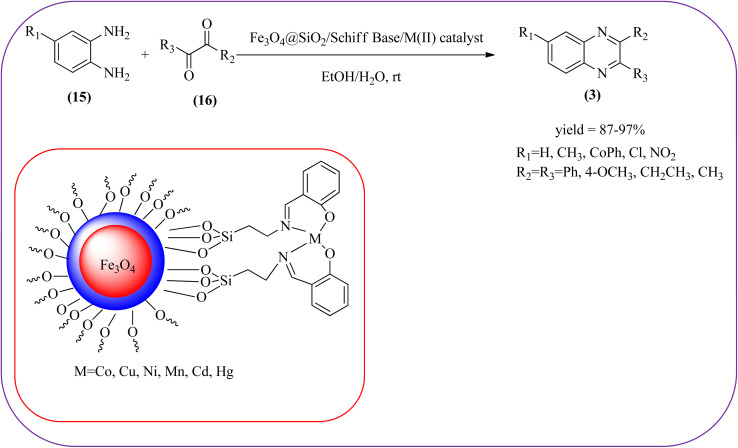
Synthesis of quinoxalines catalyzed by Fe_3_O_4_@SiO_2_/Schiff base complex of metal ions.

**Scheme 3:**
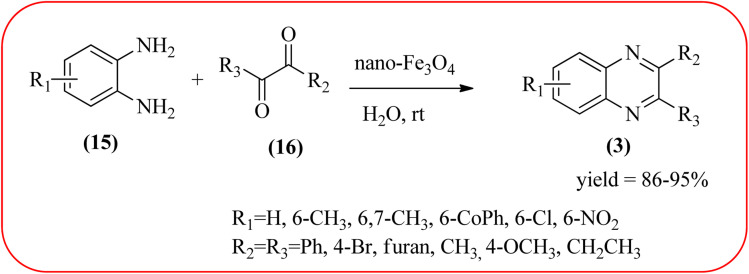
Synthesis of quinoxalines catalyzed by nano-Fe_3_O_4_ in aqueous media.

Sardarian *et al.* reported a Fe_3_O_4_@SiO_2_/Schiff base complex of metal ions catalyzed the synthesis of quinoxalines using OPD and 1,2-diketones in aqueous media at room temperature (rt). The OPD (1 mmol) and benzil (1 mmol) were chosen as the model reaction to optimize the reaction conditions performed in the presence of 0.03 g Fe_3_O_4_@SiO_2_/Schiff base/Co(ii) nanocatalyst at rt in different solvents, such as acetonitrile, chloroform (CDCl_3_), dichloromethane, ethyl acetate, water, ethanol, methanol, and different mixtures of EtOH/H_2_O (3/1, v/v). The optimized results for the synthesis of 2,3-diphenyl quinoxaline (95% yield) were obtained using ethanol EtOH/H_2_O at rt. The reaction was extended to various aromatic (electron-donating, electron/withdrawing) and aliphatic 1,2-diamines by reacting with benzil in the presence of Fe_3_O_4_@SiO_2_/Schiff base/Co(ii) (0.03 g) at rt to form various quinoxalines. The phenyl ring of 1,2-diamine with electron-donating groups (EDG), favored the formation of quinoxalines. The electron-withdrawing groups (EWG) such as –nitro, –benzoyl, and –chloro gave slightly lower yields. Especially substrate bearing –NO_2_ group gave a lower yield even after longer reaction times. The aliphatic 1,2-diamines afforded the corresponding quinoxaline derivatives in slightly lower yields and longer reaction times. In the case of 1,2-diketone, EDG associated with aromatic decreased the product yields and the effect is contrary to EWG. The author examined the catalytical activity of different metal ions like Co(ii), Mn(ii), Ni(ii), Cu(ii), Cd(ii), and Hg(ii). Among various Lewis acids, Co(ii) showed good catalytical activity and was found to be of the order Co(ii) > Cu(ii) > Ni(ii) > Mn(ii) > Cd(ii) > Hg(ii). Further, the used Fe_3_O_4_@SiO_2_/Schiff base/Co(ii) catalyst was recovered and reused five times with 97%, 96%, 96%, 95%, and 94% yield without loss of catalytical activity (Scheme 2).^88^ In the same array, Zhang *et al.*, used the magnetic Fe_3_O_4_ nanoparticles for the synthesis of quinoxaline derivatives by condensation between 1,2-diamines and 1,2-dicarbonyls. The prepared nanoparticles were round in shape, with an average diameter of 20 nm. For the optimization of solvents, the reactions were carried out with various solvents such as toluene, dichloromethane, acetonitrile, ethyl acetate, ethanol, and water. The best conversion was observed when the reaction was performed in water at 10 mol% of Fe_3_O_4_ catalyst. A similar reaction condition was used for the synthesis of various quinoxalines, the catalyst was easily recoverable by applying an external magnet and was subsequently employed in the following five cycles without a noticeable decrease in the product yield (Scheme 3).^89^

**Scheme 4:**
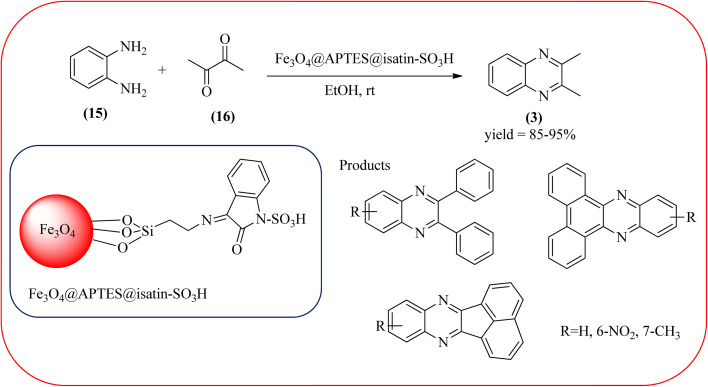
Application of Fe_3_O_4_@APTES@isatin nanocatalyst for quinoxaline synthesis.

**Scheme 5:**
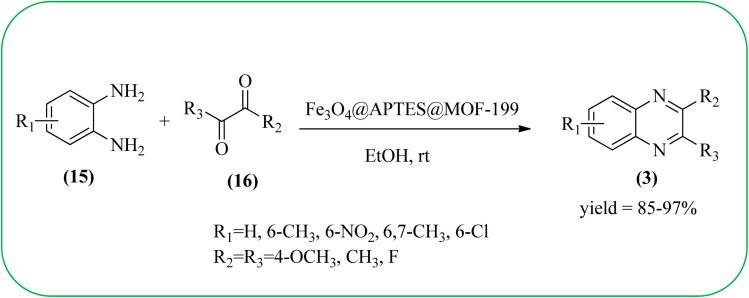
Synthesis of quinoxaline using Fe_3_O_4_@APTES@MOF-199 nanocatalyst.

**Scheme 6:**
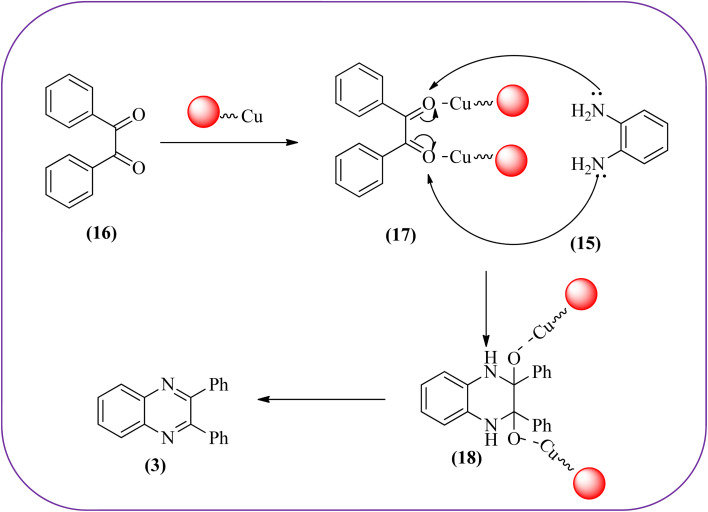
Possible mechanism for synthesis of quinoxaline using Fe_3_O_4_@APTES@MOF-199 nanocatalyst.

Sajjadifar *et al.* reported the preparation of Fe_3_O_4_@APTES@isatin catalyst through immobilization of isatin sulfonic acid on silica-modified Fe_3_O_4_ nanoparticles that was shown to be an efficient heterogeneous catalyst for the synthesis of quinoxaline derivatives using ethanol at rt. These synthesiszed nano particles have nearly spherical shape with sizes of about 16 nm. The reactions are optimized using different solvents, catalyst loading, and temperature through the reaction between OPD (1 mmol) and benzyl (1 mmol) as the model reaction. The desired product was obtained in good yield using 25 mg of the catalyst and ethanol as a solvent. The catalyst was recycled and reused seven times without loss of activity, and it was indicated that Fe_3_O_4_@APTES@isatin was a superior catalyst. The 1,2-dicarbonyl compounds including, benzil, phenanthrene-9,10-dione, and acenaphthylene-1,2-dione were reacted with OPD with electron-releasing/electron-withdrawing substituents to furnish the corresponding products (Scheme 4).^90^ In continuation, the author prepared Fe_3_O_4_@APTES@MOF-199 magnetic nanoparticles and characterized by Fourier transform infrared spectroscopy (FTIR), powder X-ray diffraction (XRD), scanning electron microscopy (SEM), energy dispersive X-ray (EDX), vibrating sample magnetometer (VSM) and thermogravimetric analysis (TGA). The particle size of the nanocatalyst is about 15–96 nm and the prepared catalyst was used for the synthesis of quinoxaline derivatives using ethanol as a solvent at rt. The author claimed the merits of the protocol are green catalyst, economic, easy workup, eco-friendly, non-toxic, and nanocatalyst with magnetic properties which can be easily recovered by a simple magnet, and can be reused several times (Scheme 5). The plausible mechanism of the reaction is proposed and the reaction pathway involves an initiation step, in that the carbonyl groups in diketone (16) are activated by nanocatalyst and further react with OPD (15) forms amino-1,2-diol (18) followed by dehydration to give quinoxalines (3) (Scheme 6).^91^

**Scheme 7:**
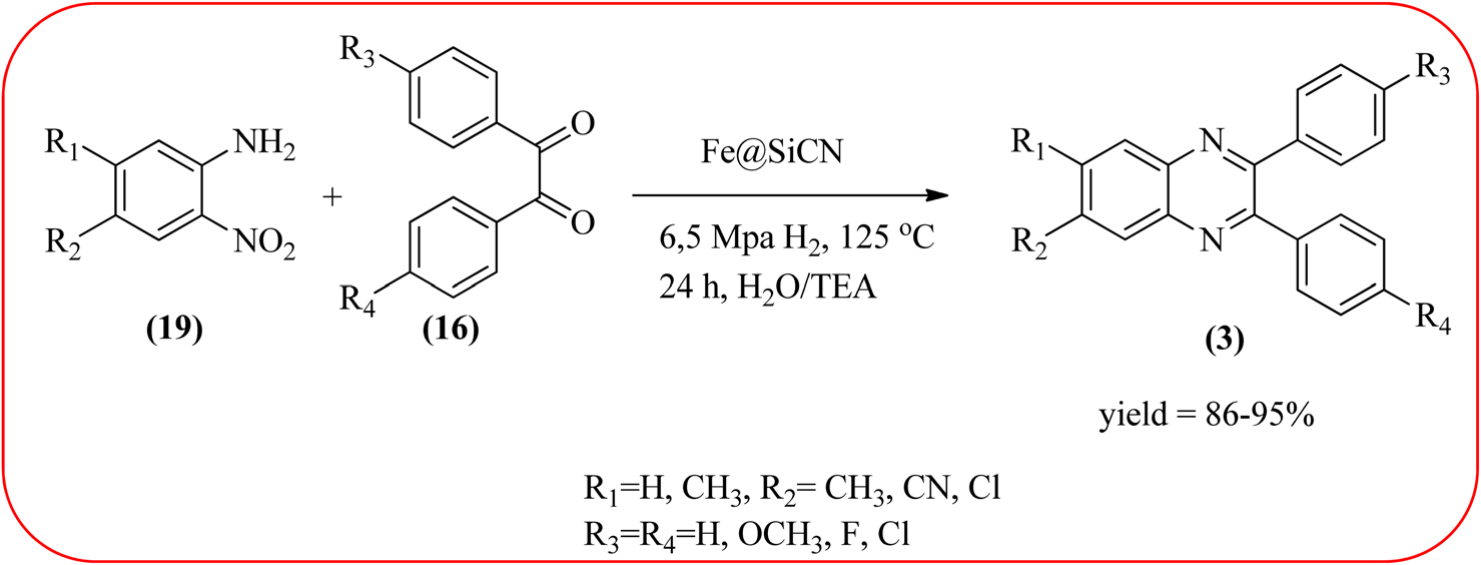
Synthesis of quinoxaline using Fe@SiCN catalyst.

**Scheme 8:**
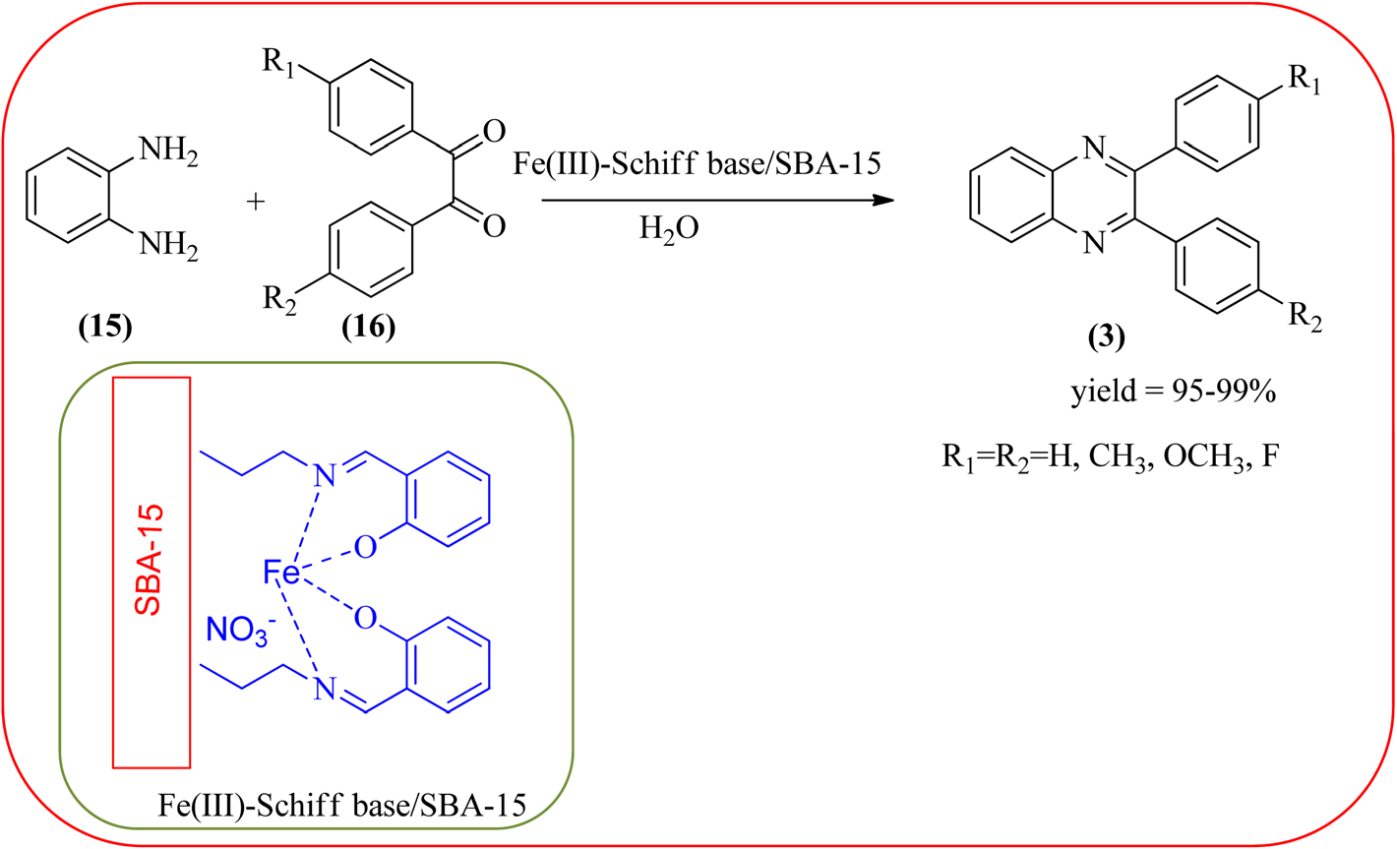
Synthesis of quinoxaline heterocycles catalyzed by Fe(iii)-Schiff base/SBA-15 in water.

**Scheme 9:**
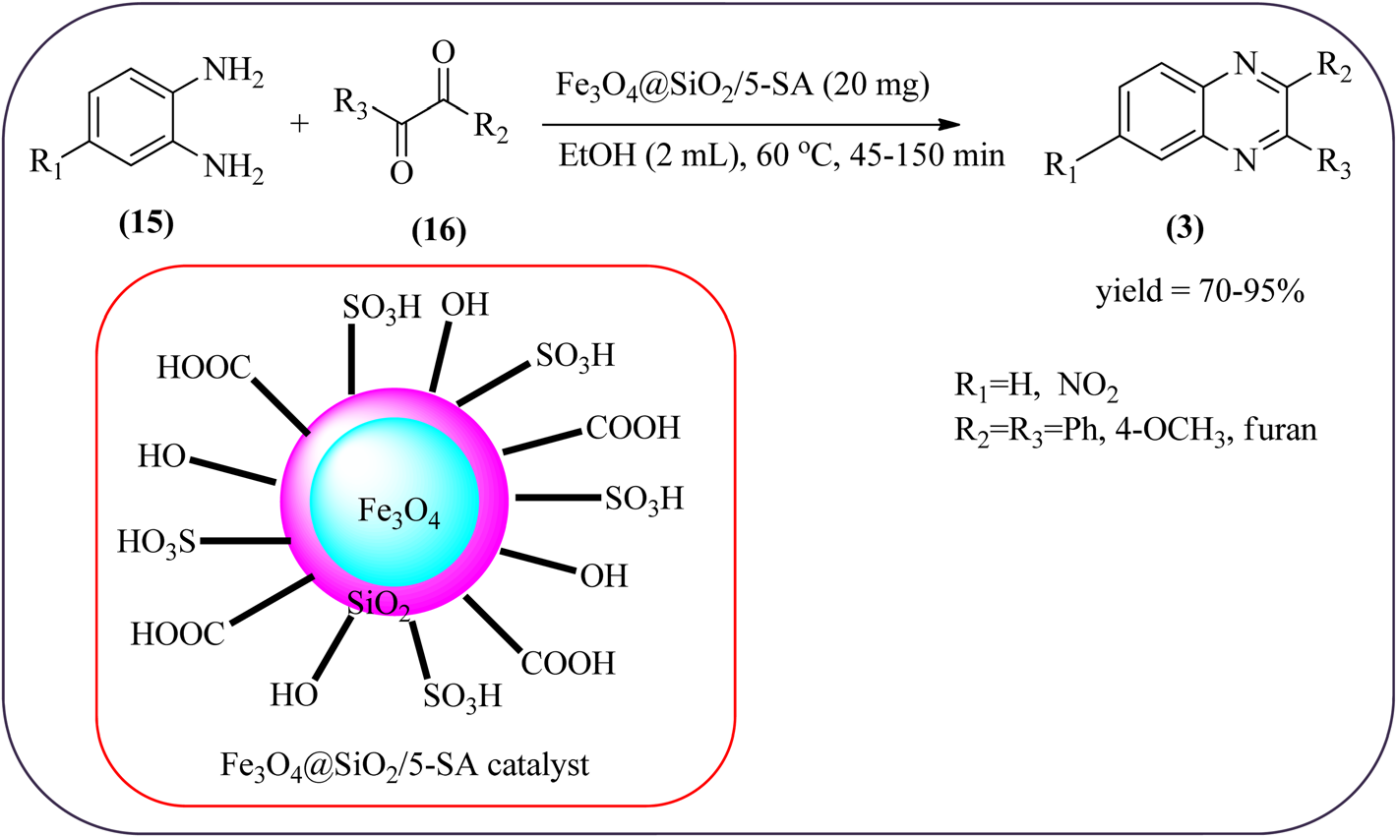
Application of Fe_3_O_4_@SiO_2_@5-SA catalyst for the synthesis of quinoxalines.

The replacement is an attractive and selectivity pattern for the development of novel coupling reactions. Using the same concept, Kempe *et al.*, have synthesized quinoxaline derivatives from nitroarenes (19) and diketones applying silicon carbonitride-based iron (Fe@SiCN) as a catalyst. This catalyst is homogeneously distributed particles with an average particle size of 5 nm. The advantages of the protocols are robust, reusable, easy to synthesize, and easy to handle iron catalyst (Scheme 7).^92^ Malakooti and co-workers synthesized Fe(iii)-Schiff base/SBA-15 heterogeneous nanocatalyst from Schiff base complex was encapsulated in SBA-15 mesoporous silica. From TEM image author confirmed this catalyst is supported complex shows retention of the hexagonal structure following grafting of the complex onto SBA-15. The catalyst is applied for the synthesis of pyridopyrazine and quinoxaline heterocycles. The catalyst showed good activity and high selectivity yield and was easily recycled taking advantage of the magnetic property (Scheme 8).^93^ Azizi's group developed a magnetic nanoparticle with high multifunctional acidic groups by anchoring water-soluble 5-sulfosalicylic acid onto the surface silica-modified Fe_3_O_4_ (Fe_3_O_4_@SiO_2_@5-SA) for the synthesis of quinoxaline derivatives from direct condensation of substituted 1,2-diamine with various 1,2-dicarbonyl in ethanol at rt with excellent yield. The catalyst sizes of the MNPs were not precisely identical and showed core–shell morphology in various sizes, constructed by some nanoparticles and the size of the MNPs particles was well below 80 nm. After the reaction, the catalyst can be recovered by an external magnet and reused for five recycles without the effect of catalytical activity. Further, the author studied the effect of substitutes on diketone as well as for diamino compounds. For the aromatic 1,2-diketones with EWG or EDG, no notable difference in yields and reaction time was observed. In the case of 1,2-diamino benzenes, the EWG (like –NO_2_), deactivated the aromatic ring and gave the product in moderate yields at prolonged reaction time. But it is the opposite for the EDGs, which were found to improve the rate of reaction and furnished quinoxaline derivatives in excellent yields (Scheme 9).^94^

**Scheme 10:**
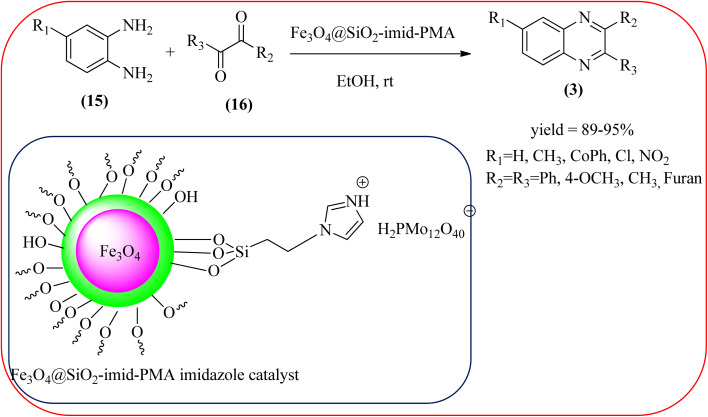
Fe_3_O_4_@SiO_2_-imidazole-PMA magnetic porous nanosphere catalyzed synthesis of quinoxaline.

**Scheme 11:**
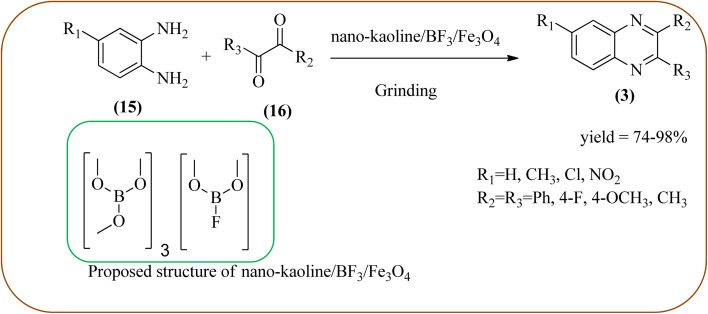
Synthesis of quinoxaline using nano-kaoline/BF_3_/Fe_3_O_4_ nano-catalyst.

**Scheme 12:**
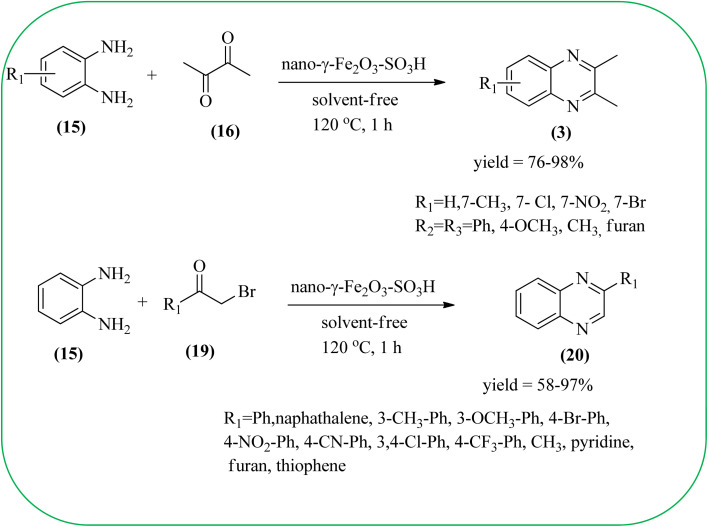
Synthesis of 2,3-diaryl/heteroaryl/2-aryl/heteroaryl quinoxalines using nano-γ-Fe_2_O_3_–SO_3_H catalyst.

**Scheme 13:**
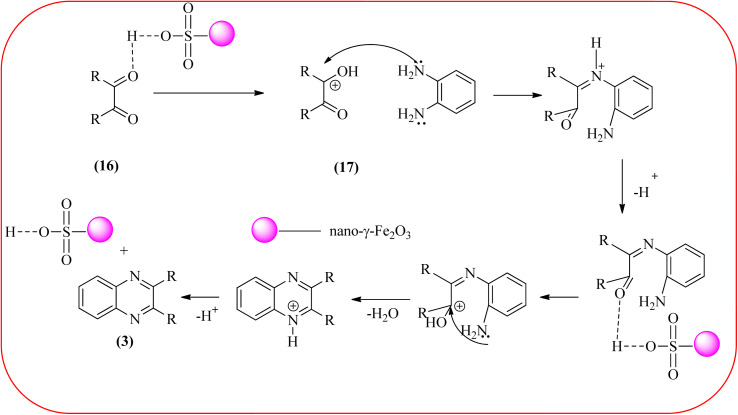
Proposed mechanism for the formation of quinoxalines using nano-γ-Fe_2_O_3_–SO_3_H catalyst.

Javidi *et al.* explored the activity of Fe_3_O_4_@SiO_2_-imidazole-PMA magnetic porous nanosphere for the synthesis of quinoxaline derivatives. The prepared nanoparticles have spherical shapes with approximately 50 nm diameters and the size distribution of these is centered at a value of 55 nm. The author observed the electronic effect of the substrate affects the yield of the products. The EDGs at the phenyl ring of amine favored the formation of the product. Whereas, amine with EWGs such as –nitro, –benzoyl, and –chloro, gave slightly lower yields. The aromatic ketone with EDG decreased the product yields and the effect is contrary to EWG. After the completion of the reaction, the magnetic catalyst can be easily separated by the external magnetic field and reused for six times without loss of efficiency. The merits of the protocol are short reaction times, nanocatalyst stability, mild reaction conditions, excellent yields, simple work-up procedure, and involvement of an efficient and recyclable catalyst (Scheme 10).^95^ Mirjalili *et al.* studied the catalytic activity of nano-kaoline/BF_3_/Fe_3_O_4_ nano-catalyst, used for quinoxaline preparation *via* condensation of 1,2-phenylene diamines and 1,2-diketones under grinding conditions. Kaoline is a clay mineral consisting of hydrated aluminum silicate and the particle size of the catalyst below 25 nm. The author claimed the advantages of the methodologies such as being solvent-free, good to excellent yields, easy work-up; and using non-toxic, inexpensive, and reusable catalysts (Scheme 11).^96^ Rangappa *et al.* used the heterogeneous nano-γ-Fe_2_O_3_–SO_3_H catalysis for the synthesis of quinoxalines using OPDs with electronically diversified 1,2-diketones and α-bromoketones *via* simple cyclo-condensation reaction under solvent-free conditions. The OPDs with EDG afforded quinoxalines in good yield when compared with OPDs with EWG. The α-bromoketone without substitution favored the formation of quinoxalines in higher yield compared to the substituted α-bromoketone. The catalyst was recovered and reused for five runs without any deactivation. The author proposed a mechanism, it involves the protonation of carbonyl groups of 1,2-diketone followed by cyclo-condensation with OPD (Schemes 12 and 13).^97^

**Scheme 14:**
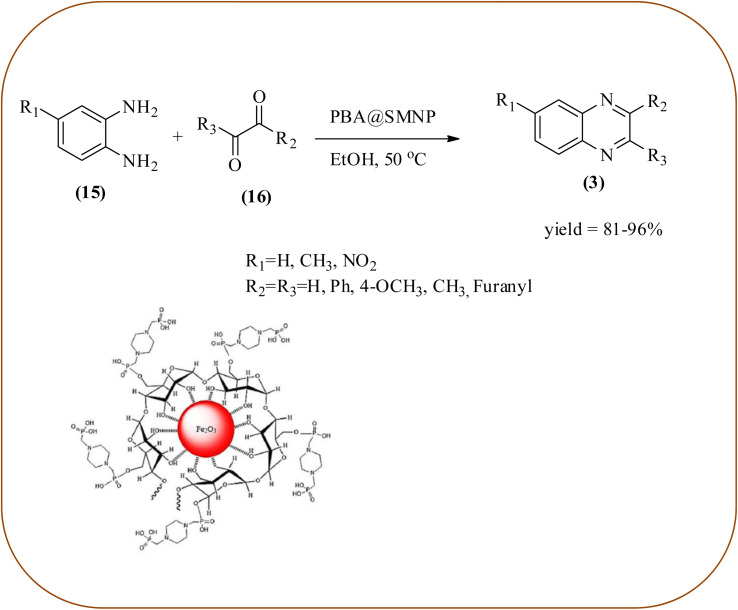
Synthesis of quinoxalines over PBA@SMNP.

**Scheme 15:**
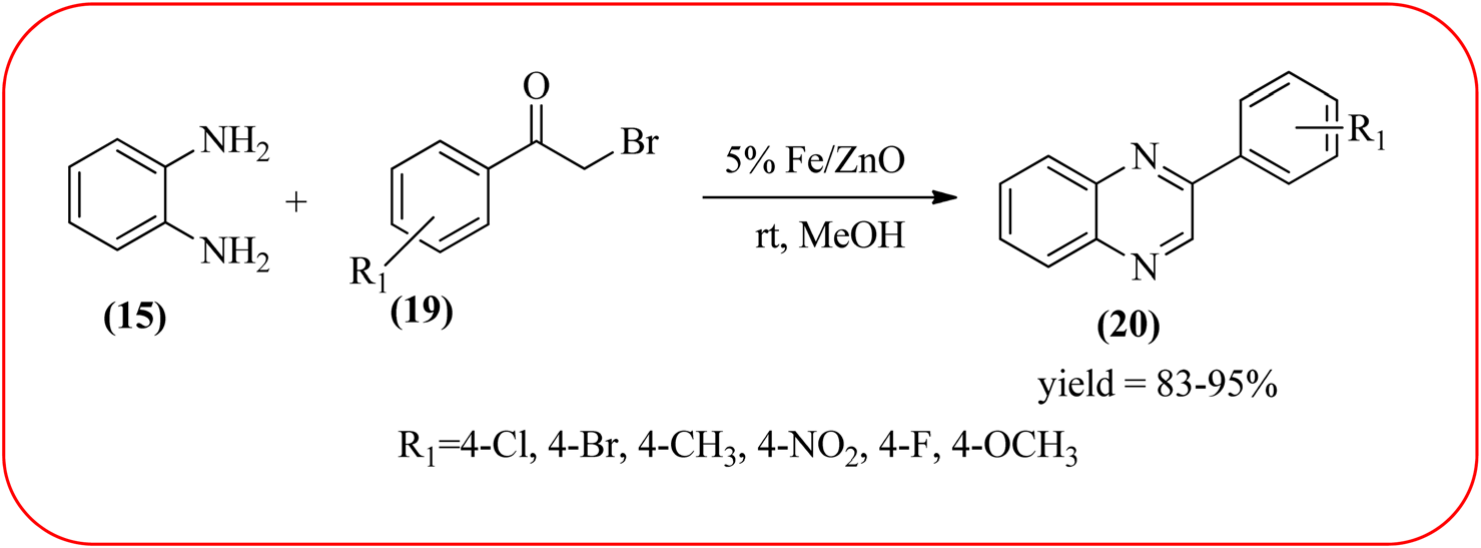
Synthesis of quinoxalines using 5% Fe/ZnO nano particles.

**Scheme 16:**
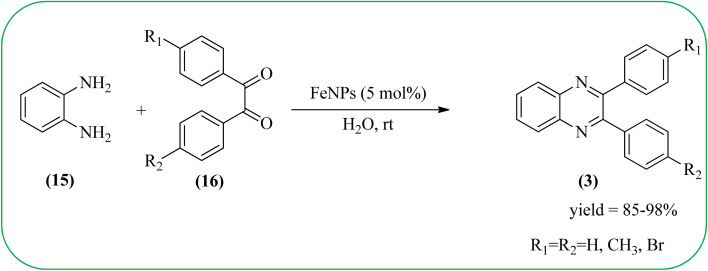
Synthesis of various quinoxalines catalyzed by FeNPs.

**Scheme 17:**
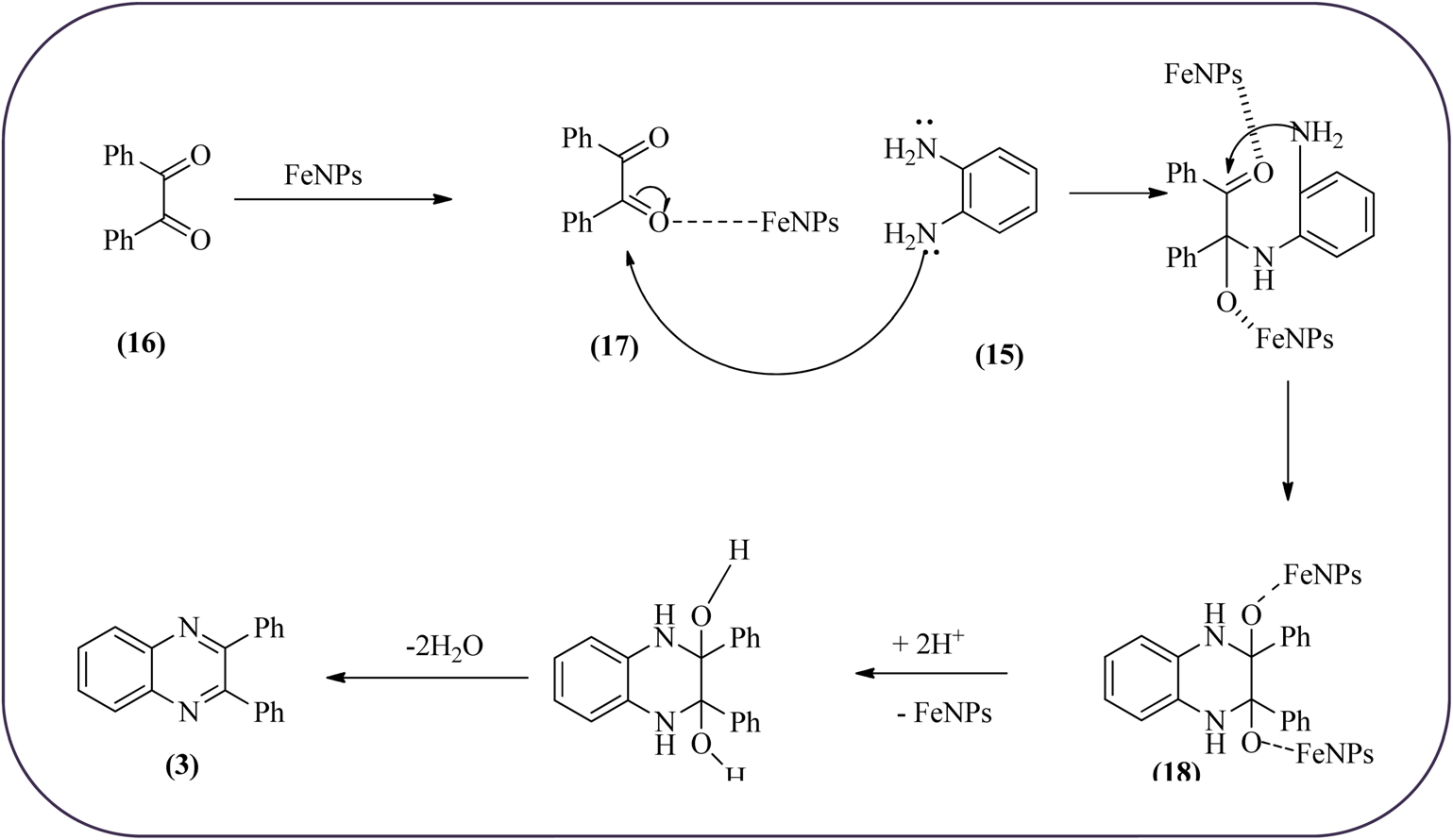
Plusible mechanism for the synthesis of quinoxalines using by FeNPs.

Farrokhi and co-worker synthesized inorganic–organic hybrid nanocomposite 1,4-piperazinediylbis(methylene) bisphosphonic acid (PBA) with starch-coated maghemite nanoparticles (SMNP) (PBA@SMNP) by supporting a bisposphonic acid on starch-coated g-Fe_2_O_3_ nanoparticles. These materials were characterized by IR, XRD, SEM, TEM, and VSM. The materials appeared as spherical or quasi-spherical shapes of PBA@SMNP nanocomposite particles, with an average diameter of 11 nm. The catalytic activity of this material was evaluated using 1,2-diamines with 1,2-dicarbonyls and aldehydes to get the desired benzimidazoles and quinoxalines respectively. The author concluded; the presence of Brønsted acid organocatalyst on the surface of maghemite nanoparticles assured their stability and has a considerable effect on their efficiency. The catalyst can be recovered using a magnet and no deactivation occurred on the catalyst after it was reused for 5 runs (Scheme 14).^98^ Borhade *et al.*, used a nanocrystalline 5% Fe/ZnO catalyst for the preparation of quinolones from OPDs and phenacyl bromides at rt. The nanomaterial is prepared from the hydrothermal method and characterized by XRD, SEM, EDAX, TEM, and SAED techniques. The TEM analysis reveals that the nanoparticles are hexagonal with several spherical-shaped crystallites and the average size of nanocrystallites is around 62.3 nm. From the electronic substitution study, the phenacyl bromide with EDG furnishing gave corresponding quinoxaline, high yield with less time but it is reversed in the case of EWG substituents. After the recovery of the catalyst, it is stable and reusable even after five cycles without appreciable loss in activity (Scheme 15).^99^ Arde and co-workers synthesized amorphous iron nanoparticles (FeNPs) using an aqueous leave extract of *Boswellia serrata* plant and the FeNPs were stabilized *in situ* by the addition of aqueous extract of *Acacia concinna* as a bio-surfactant. These NPs were characterized using XRD, UV, EDX, SEM, TEM, and other techniques. These nanoparticles are amorphous in nature with particle size around 19 nm. The catalytical activity of these FeNPs was performed by the synthesis of quinoxalines using OPDs with electronically diversified benzils. The benzils without any substituent and substituents with EWGs gave the desired quinoxalines in a shorter time with good yield when compared with benzil with EDG, which took a long time. The mechanism of the reaction proceeds by oxidative addition of FeNPS on carbonyl oxygen of benzil followed by nucleophilic attack of lone pair of nitrogen of OPD on activated carbonyl carbon and the same process was followed for benzil to form a cyclic intermediate. The six-membered cyclic intermediate abstracts proton from solvent, leaving behind FeNPS to form a dihydroxy intermediate followed by dehydration to obtain the corresponding quinolaxines (Schemes 16 and 17).^100^

### 2.2. Copper-based nanocatalysts for the synthesis of quinoxalines

**Scheme 18:**
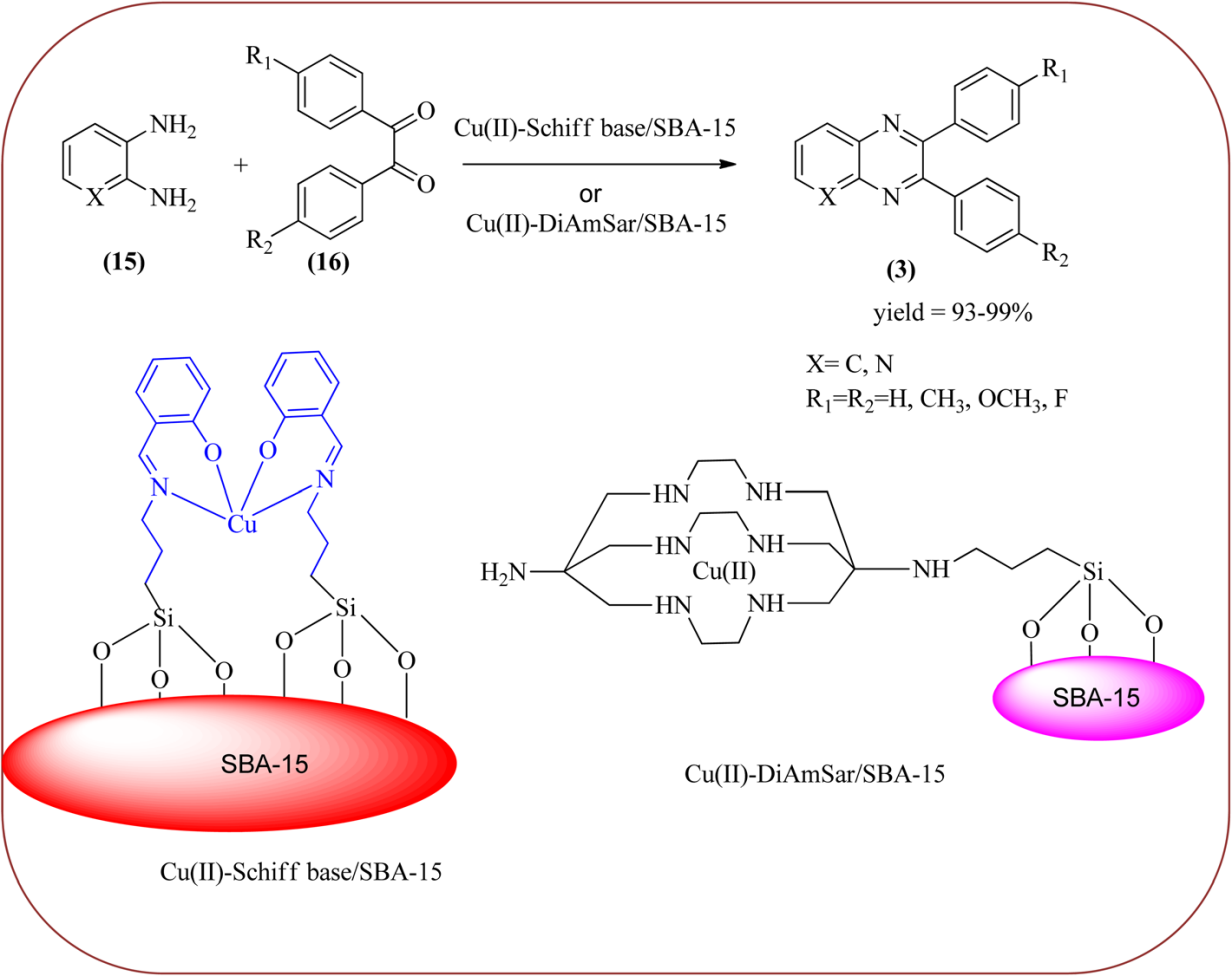
Synthesis of quinoxalines derivatives in the presence of Cu(ii)-Schiff base/SBA-15.

**Scheme 19:**
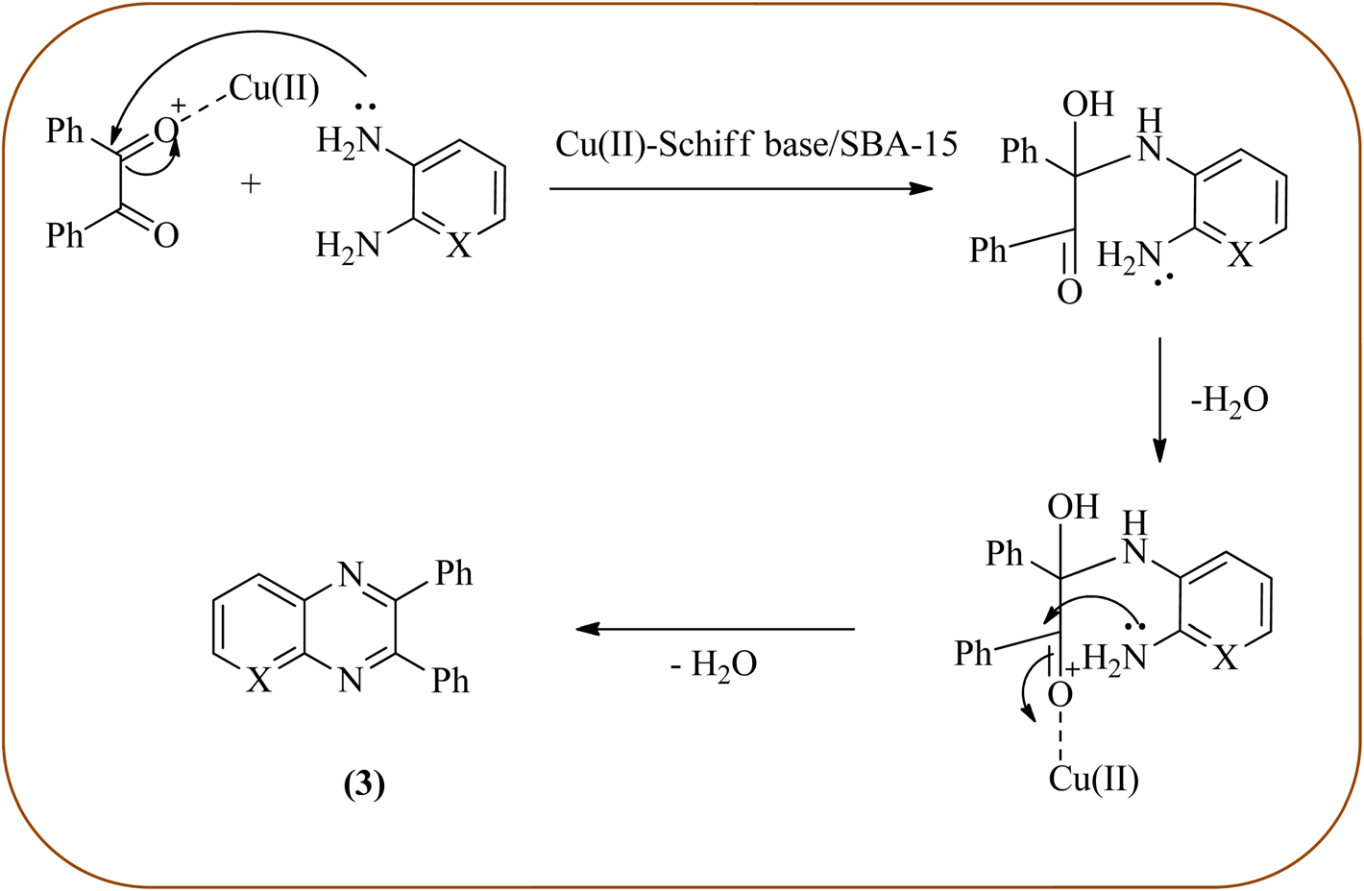
Plausible mechanism for the synthesis of quinoxalines using Cu(ii)-Schiff base/SBA-15.

**Scheme 20:**
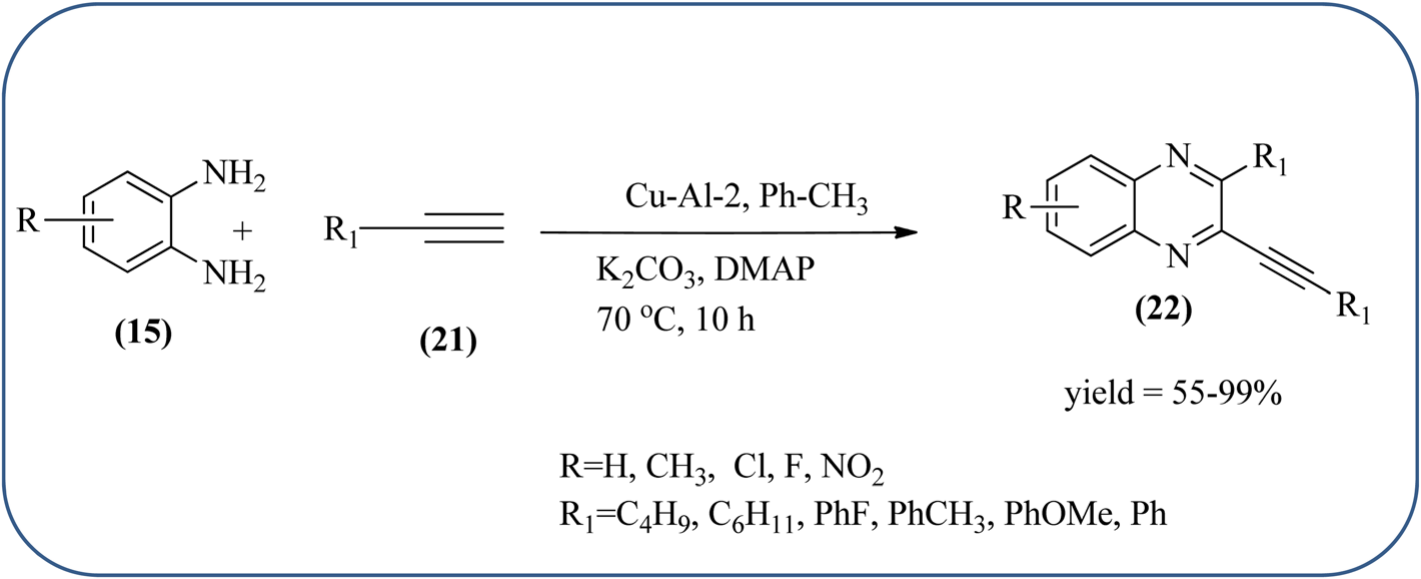
Cu–Al-2 catalyzed synthesis of quinoxalines.

Copper has a high boiling point, which makes it compatible with high-temperature and pressure chemical reactions, including microwave-assisted reactions, vapor-phase reactions, continuous flow reactions, and various organic transformations. Copper-based nanocatalysts have found many applications in nanotechnology, including catalytic organic transformations, electrocatalysis, and photocatalysis.^101–103^ Also, the copper-based nanocatalysts/nanomaterial is used for the synthesis of quinoxalines, and the same is discussed here. Bardajee and co-workers prepared Cu(ii)-Schiff base/SBA-15 catalysts and characterized them from TEM, XRD, and nitrogen adsorption/desorption isotherms. The morphology of the material showed a 2D-hexagonal array of uniform linear channels with the typical honeycomb. The catalyst exhibited lack of pore blocking or agglomeration of copper metal and the pore size can determine to be 8 nm for the catalyst. Further, this catalyst was utilized for the synthesis of quinoxaline and pyridopyrazine derivatives. The reactivity of diamines is generally dominated by electronic effects. The hetero-aromatic diamines showed less reactivity in comparison with aromatic or aliphatic diamines. The catalyst recovered after the completion of the reaction and reusability over six successive runs without the loss of activity. In the same array, the author synthesized the same quinoxaline derivatives using Cu(ii)-DiAmSar/SBA-15. This material retaining of the cylindrical shape of the pores and the hexagonal arrays of uniform channels (Schemes 18 and 19).^104,105^ Nakhate and co-workers described the synthesis of quinoxaline derivatives from various terminal alkynes with OPDs by using copper alumina catalyst. Author used different mole ratios of Cu^2+^/Al^3+^, 2 : 1 (Cu–Al-1), 2.5 : 1 (Cu–Al-2) and 3 : 1 (Cu–Al-3) for this methodology. These materials are agglomerated particles and there is no significant change in morphology was observed for the different ratios of Cu^2+^/Al^3^ (2, 2.5 and 3). Among these, Cu–Al-2 showed excellent activity at 60 °C in presence of K_2_CO_3_. From substrate study, author concluded terminal alkynes with electron-deficient groups afforded excellent yields as compared to the electron-rich species. Also, present protocol works good with aliphatic alkynes such as hexyne and cyclohexyne. Where in case OPD, electron-rich group such as methyl, afforded a good yield but the strong electron-deficient group such as –NO_2_ does not tolerate the reaction, hence no product formation. The recovered catalyst was used four times without significant loss in catalytic activity (Scheme 20).^106^

**Scheme 21:**
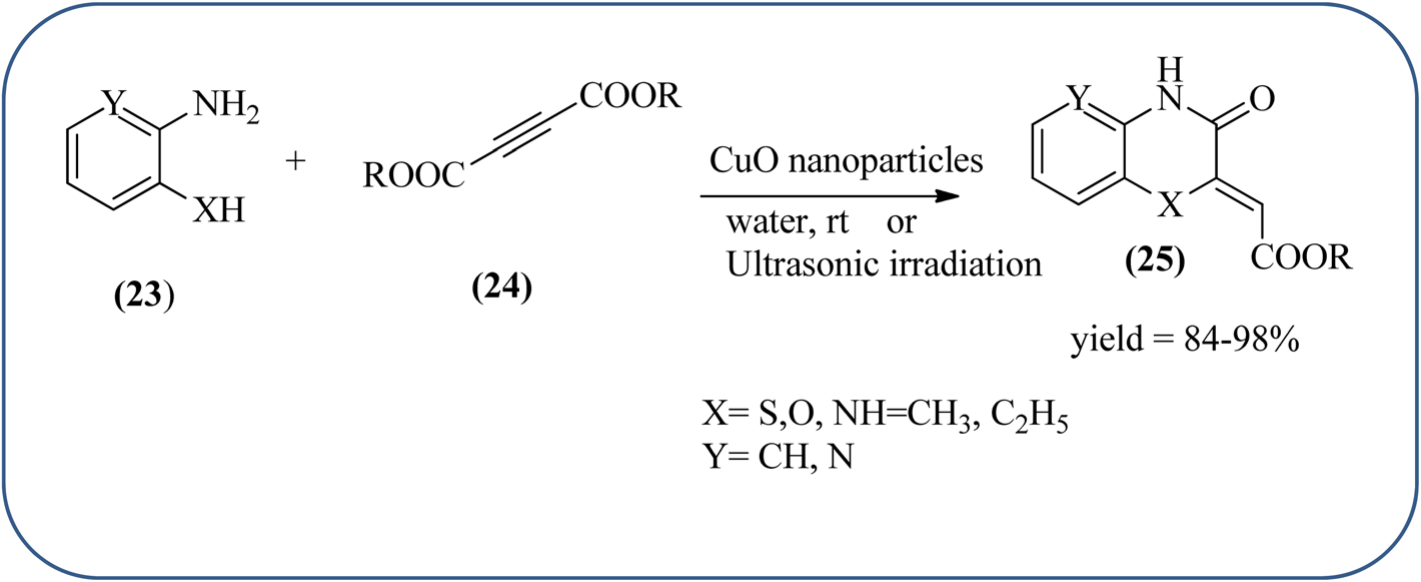
An efficient synthesis of benzoheterocycles using CuO nanoparticle.

**Scheme 22:**
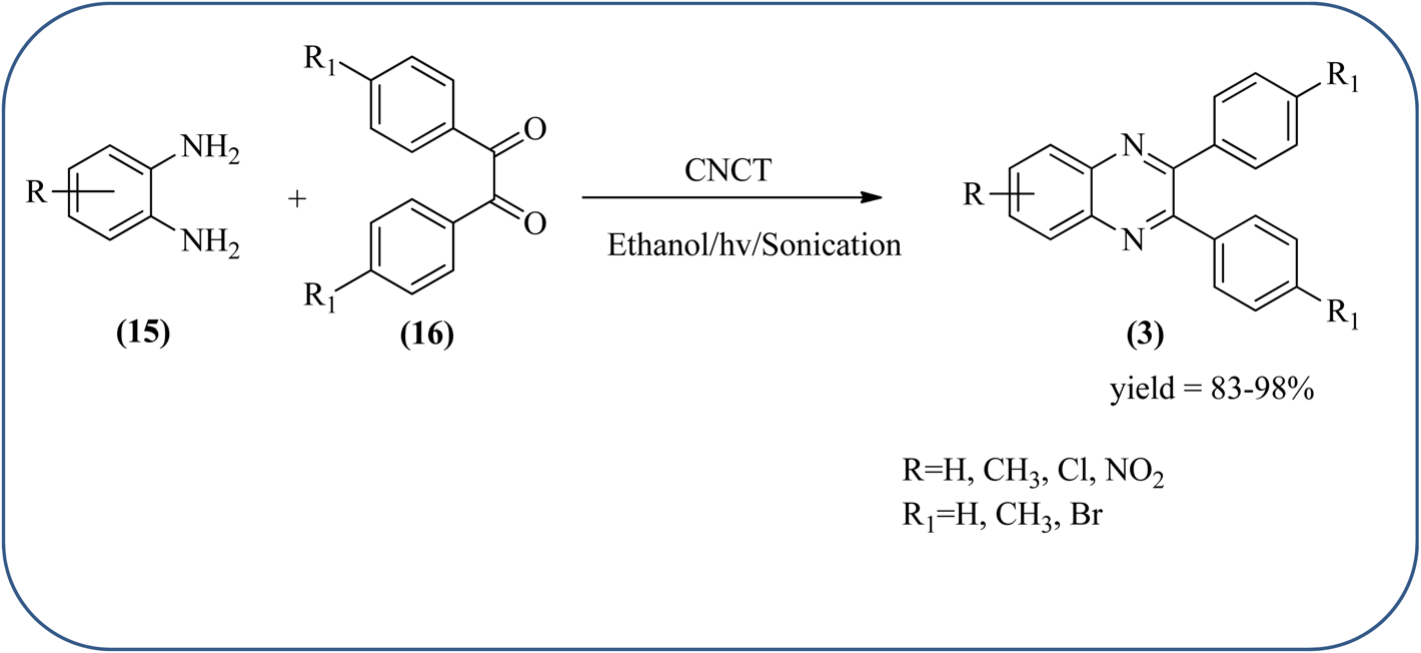
Synthesis of quinoxalines using g-C_3_N_4_/Cu_3_TiO_4_ (CNCT).

**Scheme 23:**
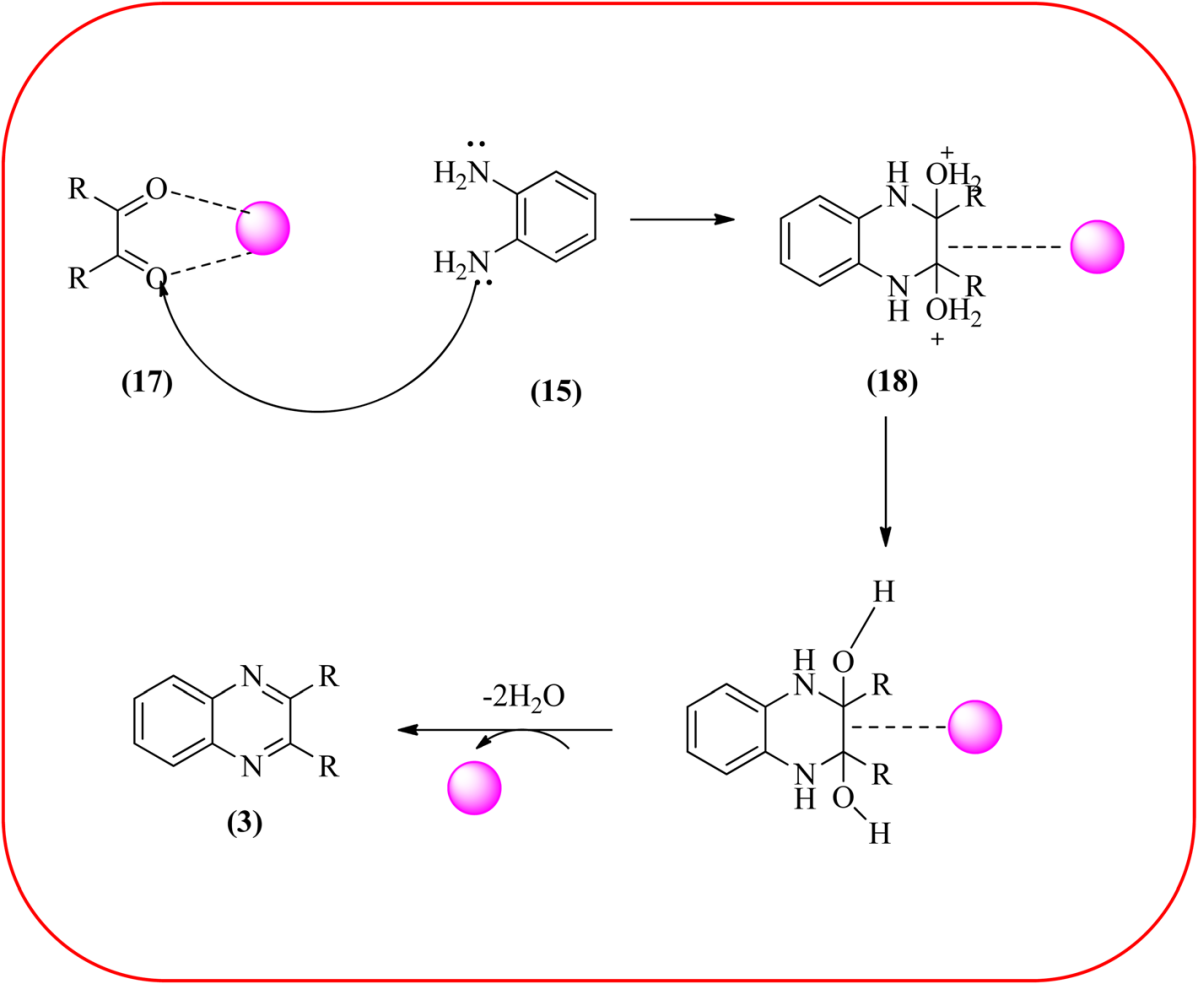
Plausible mechanism for the synthesis of quinoxalines using CNCT.

Sadjadi *et al.*, developed efficient method for the synthesis of quinoxaline, benzoxazine, and benzothiazine using nanocrystalline copper(ii) oxide. These CuO were characterized by XRD and transition electron microscopy (TEM) techniques. The average size of the obtained CuO particles is 5 nm and the mean value of surface area was 32.457 m^2^ g^−1^. The reactions were carried out at aqueous media and catalyst is recovered after the completion and reused up to four cycles without affecting the activity. In continuation, author used same catalyst and prepared various benzoheterocycles under ultrasonic irradiation. The advantages of the protocol are higher yields, short reaction times, and mild reaction conditions, with reusability of the catalyst (Scheme 21).^107,108^ Arunachalapandi and co-workers developed a heterogeneous g-C_3_N_4_/Cu_3_TiO_4_ (CNCT) catalyst for the preparation dihydro-quinazolinone and quinoxaline compounds from benzil, diamines, and aldehydes in ethanol under ultrasound with visible light irradiation. The synthesized CNCT nanocomposite was characterized by XRD, TEM, AFM, EDX, X-ray photoelectron spectroscopy, UV-vis diffuse reflectance spectroscopy, Photoluminescence, BET, zeta potential, and thermogravimetric analysis. Through these analyses it was confirmed that material/catalyst is high crystalline nature, optical light-absorbing property, high surface area, and stability. The catalyst is sphere-like morphology and particle size is around 47 nm. Through these methods various quinazolinone and quinoxaline derivatives were synthesized without the electronic affects. It was observed that sonication activates the catalyst surface and produces more amounts of active sites. The cavitation also reduces the activation energy barrier and gives the final products (Schemes 22 and 23).^109^

### 2.3. Nickel-based nanocatalysts for the synthesis of quinoxalines

**Scheme 24:**
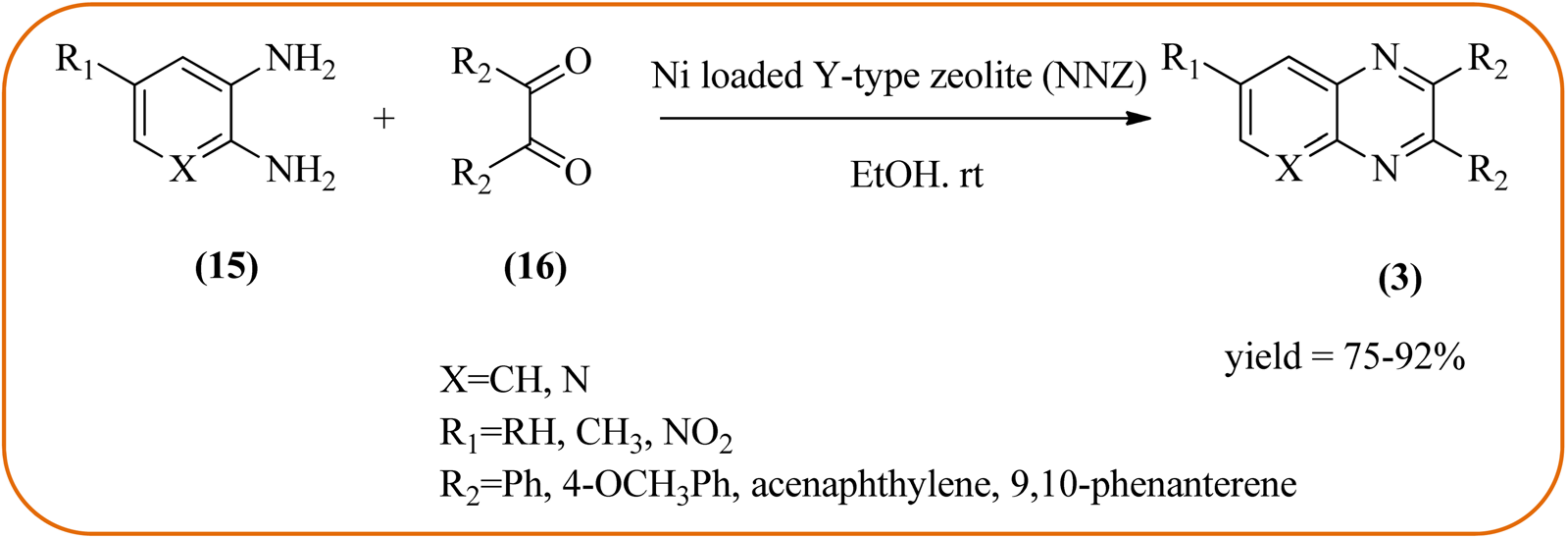
Ni(ii) ion loaded Y-type zeolite catalyzed synthesis of quinoxalines.

**Scheme 25:**
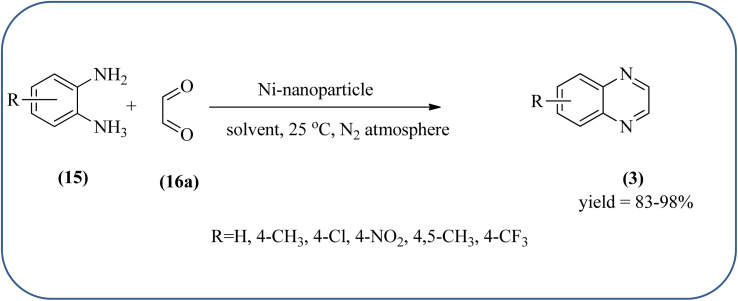
Synthesis of quinoxalines using Ni catalyst.

Nickel-based catalysts are becoming very popular in organic synthesis and are used for the synthesis of a wide range of valuable organic compounds such as spiro and condensed indole derivatives, aromatic heterocycle, 5-substituted 1*H*-tetrazoles, quinolines, spirooxindoles, polyhydroquinolines, and sulfoxidation.^110^ Various Ni-based catalysts were used for the synthesis of quinoxalines and were discussed in this section. Mehdi and co-workers developed nanoporous Ni(ii) ion-loaded Y-type zeolite (NNZ) material and applied it for the synthesis of quinoxalines, pyrido[2,3-*b*]pyrazines, and indolo[2,3-*b*]quinoxalines. The catalyst was identified with FT-IR, EDX, SEM, and BET analysis. The particle size was about 54–119 nm and the peak appeared in the region of 7.5 eV confirming the presence of nickel metal deposited on zeolite. The presence of EWG (–NO_2_) substituent on the phenyl ring diamine, decreased the reaction yield, and substituted EDG (CH_3_) was the contrary (Scheme 24).^111^ Ajeet Kumar *et al.*, synthesized monodispersed Ni-nanoparticle (size around 14–18 nm) and further utilized material for the efficient preparation of quinoxaline using a number of 1,2-diamines were condensed with glyoxal using 10 mol% of Ni-nanoparticles at 25 °C under N_2_ atmosphere. The OPD with the ED substituents the reaction was faster while EW substituents decreased the rate of the reaction. The Ni-nanoparticles play a complex role in accelerating the dehydration step of a reaction and this promotes the formation of products. The Ni-nanoparticles will be recycled by separating them from the reaction mixture by mild centrifugation and used them up to six cycles, without a change in their catalytic activity (Scheme 25).^112^

### 2.4. Gold-based nanocatalysts for the synthesis of quinoxalines

**Scheme 26:**
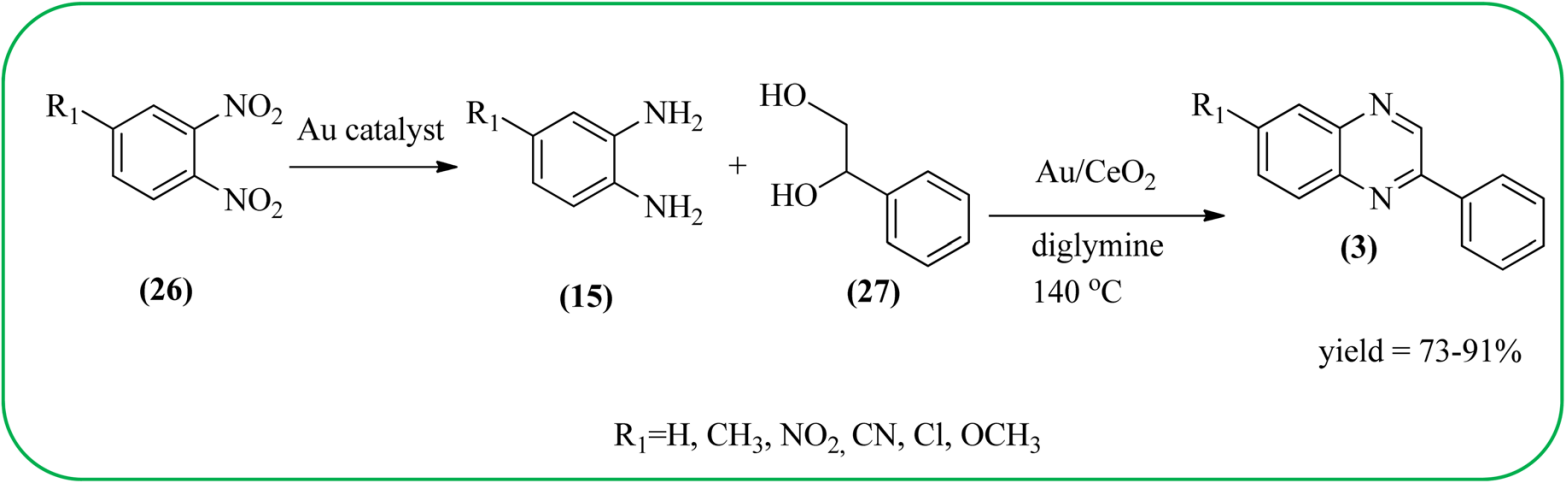
One-pot synthesis of quinoxaline derivatives using Au/CeO_2_.

**Scheme 27:**
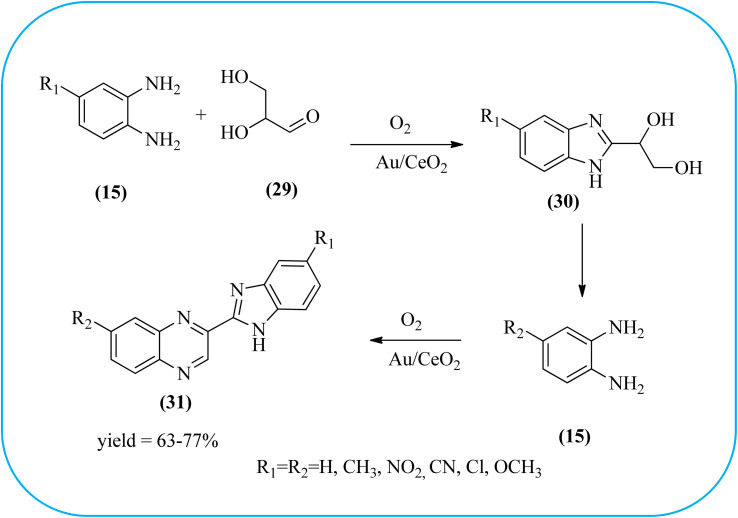
Synthesis of benzimidazoylquinoxaline using Au/CeO_2_ catalyst.

**Scheme 28:**
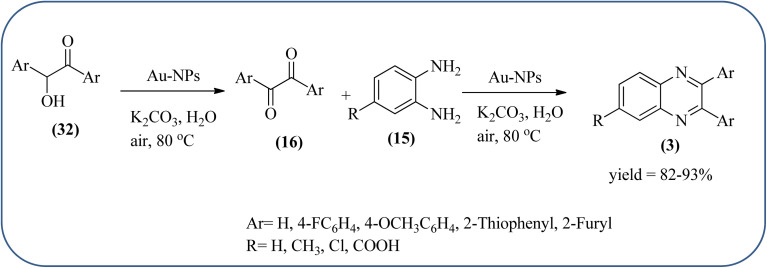
Syntheses of aryl 1,2-diketones and quinoxaline derivative using AuNPS.

The exploration of gold nanoparticles (Au-NPs) as catalysts has attracted tremendous attention in recent years in the context of developing environmentally friendly and sustainable routes to a myriad of important organic transformations. AuNPs of small size show excellent catalytic performance for many chemical reactions examples include the reduction of carbon–carbon triple bonds or of nitroarenes, aerobic oxidation of alcohols, and carbon–carbon bond formation.^113^ Also, these Au-nanoparticles are applied for the preparation of quinoxaline and its derivatives. Climent and co-workers synthesized gold nanoparticles supported by nanosized CeO_2_ (Au/CeO_2_) or hydrotalcite (Au/HT, cubic in shape) as catalysts and further used for the synthesis of quinoxaline derivatives by 1,2-propanediol and OPD under base-free conditions. The catalyst can be easily recovered and reused with a small loss of activity. The EWGs such as –nitro, –chloride, or –nitrile groups, are present, and quinoxaline derivative yields are lower with respect to OPD. The reactant with ED substituents like methyl, (–CH_2_–)_4_, or methoxy groups enhance the yield (Scheme 26).^114^ Further, the author extended the catalyst system for the synthesis of benzimidazoylquinoxaline derivatives through the oxidative coupling of glycerol or glyceraldehyde with OPD derivatives. EWGs improved the selectivity of the benzimidazoylquinoxaline compound. The catalyst could be easily recovered and reused with a small loss of activity thereby maintaining high selectivity (Scheme 27).^115^ Bhattacharya and co-workers developed 4-amino thiophenol self-assembled monolayer coated gold-nanoparticles (Au-NPs, size around 10 nm) and applied these NPs one-pot synthesis of quinoxalines *via in situ* oxidation of α-hydroxy ketones and subsequent condensation with aryl 1,2-diamines in water. The aryl α-hydroxy ketones with various substituents on the aromatic ring produced the desired oxidized product. The EWG on the aromatic ring increased the yield when compared to EDG. The reusability of the catalyst was limited after a few cycles of oxidation due to agglomeration of the nanoparticles (Scheme 28).^116^

### 2.5. Silica-based nanocatalysts for the synthesis of quinoxalines

**Scheme 29:**
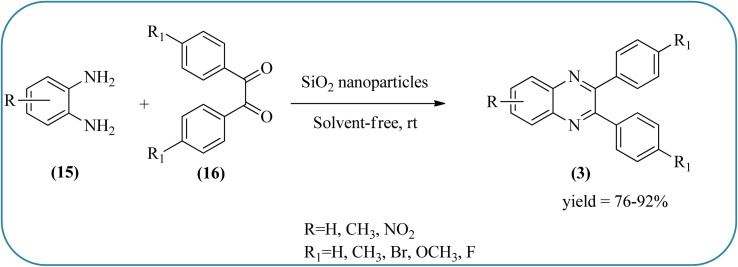
Syntheses of quinoxaline derivatives using SiO_2_ nanoparticles.

**Scheme 30:**
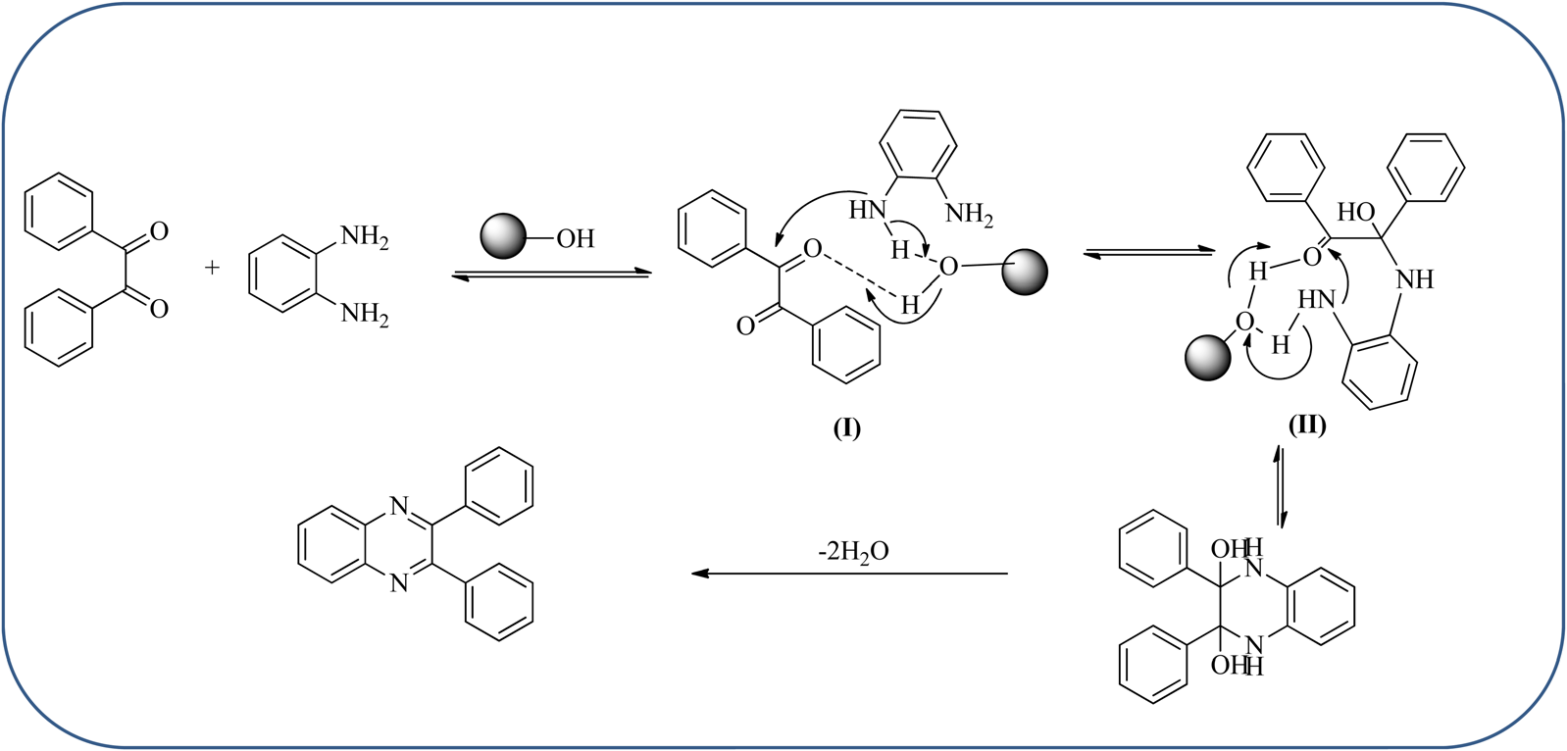
The proposed mechanism for the synthesis of quinoxalines using silica NPs.

**Scheme 31:**
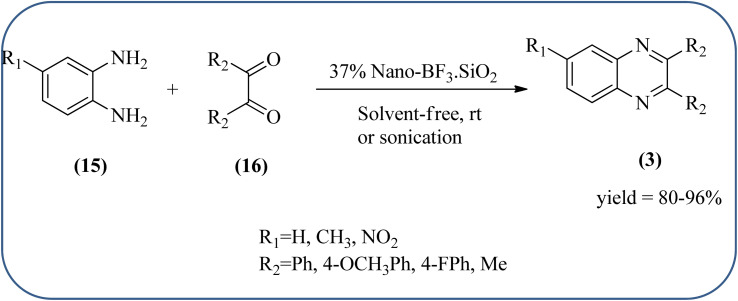
Nano-BF_3_ SiO_2_ catalyzed the synthesis of quinoxalines.

Silica nanoparticles (NPs) have aroused great attention thanks to their exclusive physicochemical properties as well as the ability to control and adjust them. These silica NPS have promising properties, such as chemical inertness, controlled porosity, high surface area, firm framework, nanometer size, and very good thermal, mechanical, and chemical stability, the metals nanoparticles coated by SiO_2_ become one of the most important catalysts of these types used in different fields. Due to the specific physicochemical characteristics of various strategic materials such as platinum (Pt), cobalt (Co), copper (Cu), palladium (Pd), and gold (Au), they are extensively utilized in many modification and synthetic strategies in modern organic synthesis, especially in catalytic activities.^117,118^ Hasaninejad and co-workers developed an efficient method for the synthesis of quinoxalines from the condensation of 1,2-diamines with 1,2-diketones by using silica nanoparticles-catalyzed under solvent-free conditions at rt and affording high yields of quinoxalines with short reaction times. The reactions were carried out with different oxides (MgO, CaO, SiO_2_ (60–120 mesh), and neutral Al_2_O_3_). The SiO_2_ nanoparticles gave excellent results in order of yield and reaction time. Highly microporous solids such as silica NPs offer a wide range of active sites and often can be regenerated if deactivated during the reaction. The aryl 1,2-diamines with EDGs had no significant effect on the reaction, whereas EWGs decreased the yields and increased the reaction times. The 1,2-diketones with different substituents have a negligible influence on the reaction yields. The presence of the reactive –OH groups on the surface of the silica NPs plays a major role in its catalytic activity. Based on this, the author proposed a plausible mechanism, the benzil is activated by the O–H group of silica NPs followed by the N-nucleophilic amine attack on the carbonyl to form intermediate I and subsequently form intermediate II. During this process, quinoxaline is produced due to dehydration. The catalyst can be recovered and reused up to fifteen times and after three cycles the slightly catalytical activity is decreased (Schemes 29 and 30).^119^ Mirjalili and co-workers have investigated nano-BF_3_ SiO_2_ as a green and reusable solid acid catalyst for the synthesis of 2,3-disubstituted quinoxalines *via* condensation of α-diketones and OPD. The nano-BF_3_ SiO_2_ is noncorrosive, eco-friendly, and reusable which has a gradual decline in activity. The reaction was carried out under solvent-free conditions at room temperature under sonication conditions (Scheme 31).^120^

**Scheme 32:**
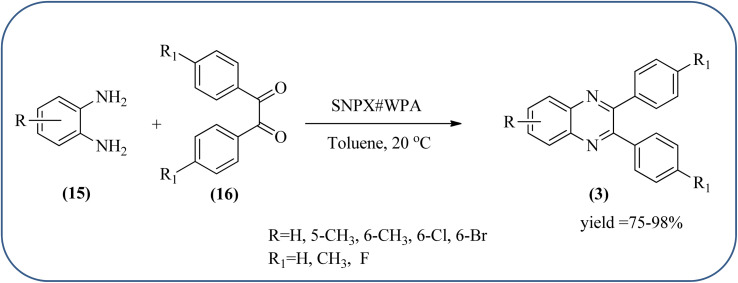
Synthesis of quinoxalines using tungstophosphoric acid immobilized on mesoporous silica nanoparticles (SNPX#WPA).

**Scheme 33:**
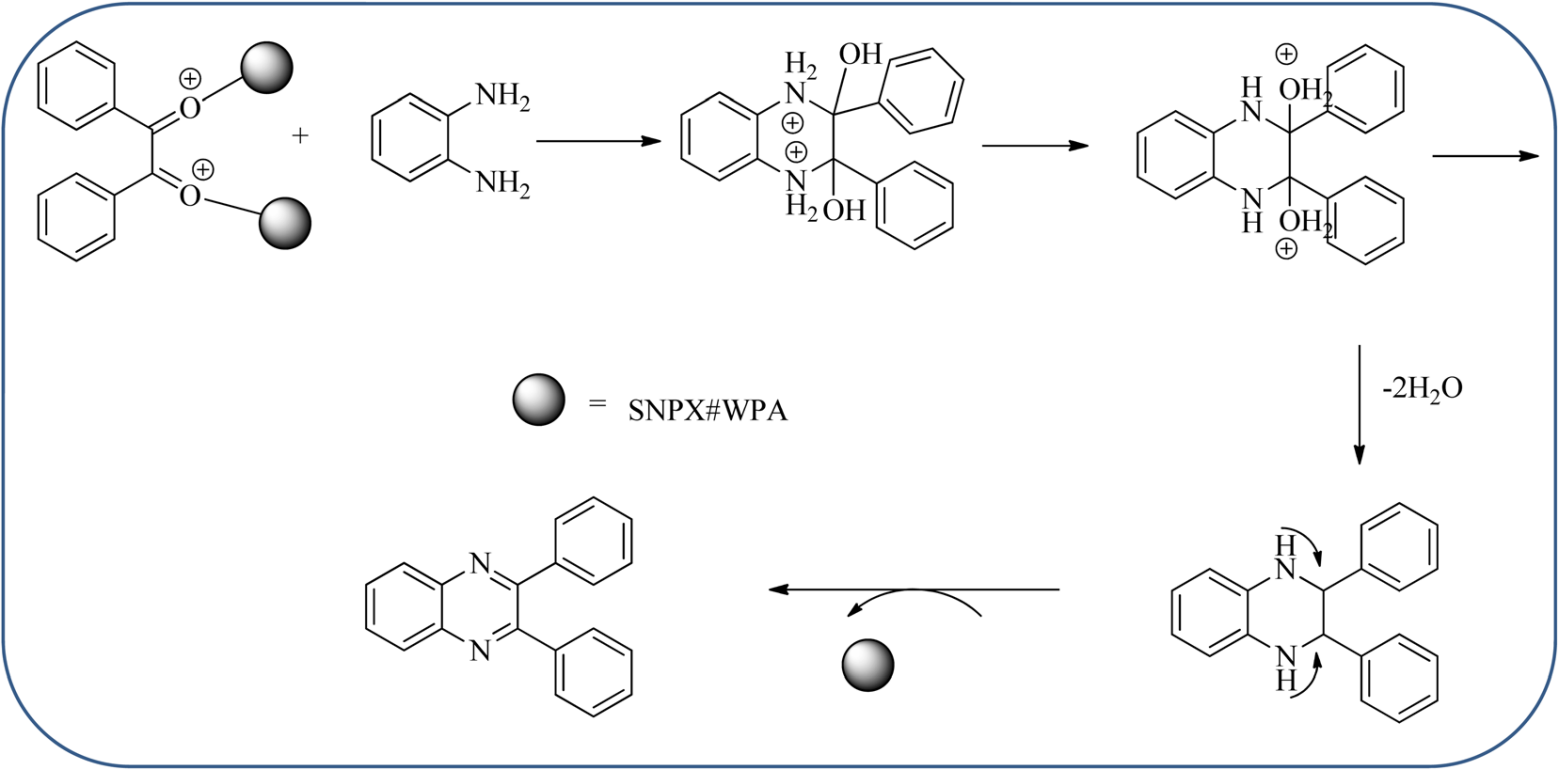
Plausible mechanism for the synthesis of quinoxalines using tungstophosphoric acid immobilized on mesoporous silica nanoparticles (SNPX#WPA).

**Scheme 34:**
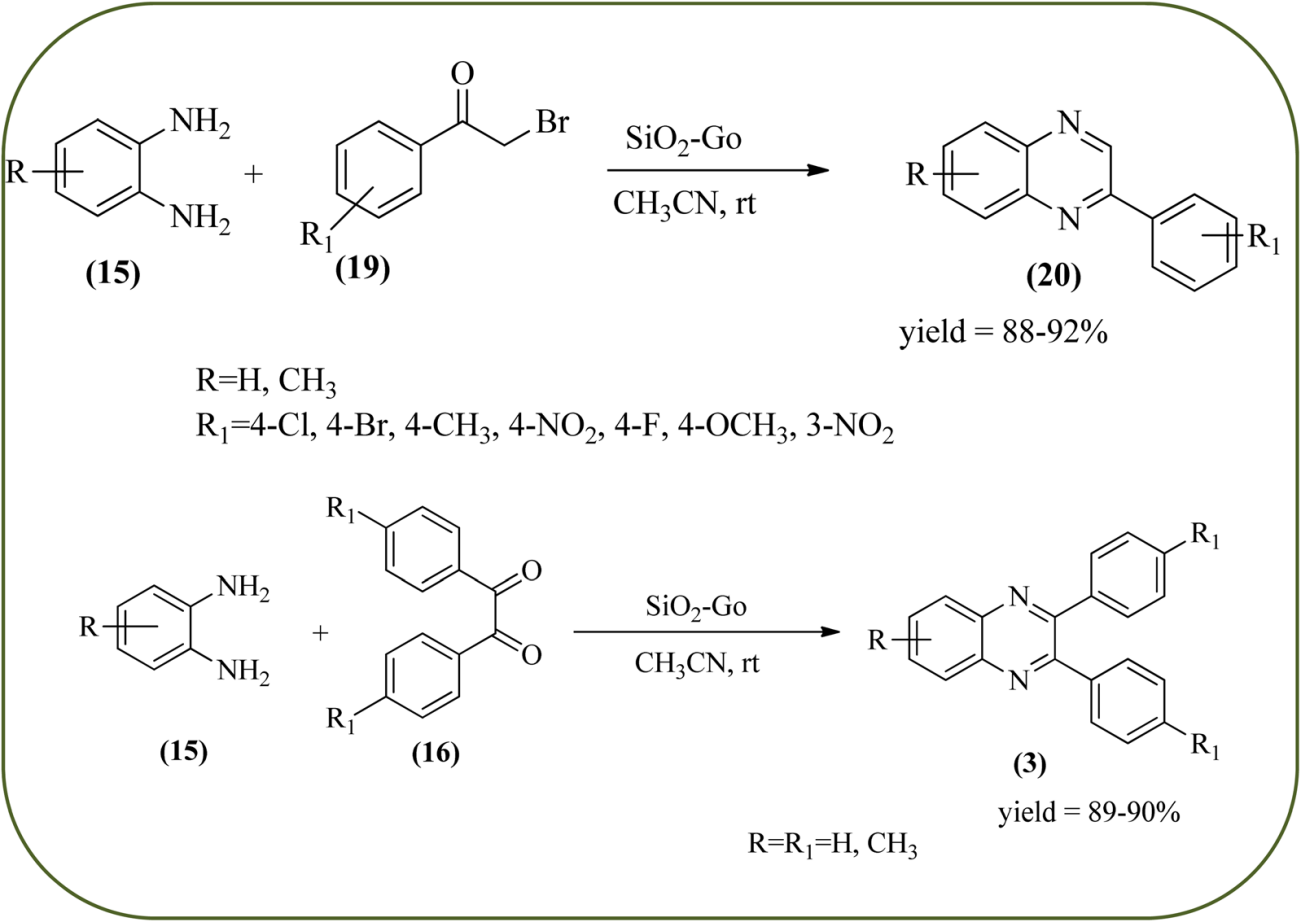
Synthesis of quinoxalines using SiO_2_-GO as a catalyst.

Sosa *et al.*, prepared an acid catalyst (nanostructure) composed of tungstophosphoric acid immobilized on mesoporous silica nanoparticles. The catalyst with the strong acid sites and specific surface area of 222 m^2^ g^−1^, mesopores centered at 8.6 and 37.0 nm, and an acidity of 194 mV. Further utilized this material, for the synthesis of quinoxaline and their derivatives by the condensation of 1,2-dicarbonyl compounds with aromatic 1,2-diamines in an aprotic organic solvent at low temperature. The presence of electron-attracting group in OPD, such as a halogen increases the yield and reduces the time to form quinoxaline with respect to non-substituted OPD. This effect is more remarkable when the aromatic ring presents a heteroatom such as nitrogen. Moreover, the introduction of a methyl group at the *ortho* position, causes an increase in the yield. The substituents in the 1,2-ethanodione with the presence of both electron-attracting (fluorine) and electron-releasing (methyl) substituents causes a significant increase in yield of the quinoxaline. Author proposed the plausible mechanism, in the first step the dione is coordinated to the catalyst, followed by a nucleophilic attack in the carbonyl carbon by the 1,2-phenylenedimine amino groups. Then, the carbocation intermediate is obtained by dehydration and elimination of the protons to give the quinoxaline, and the catalyst is regenerated (Schemes 32 and 33).^121,122^ The silica-based nanosphere-graphene oxide (SiO_2_-GO) hybrid was prepared by Shitre and co-workers. Further, this material was characterized by TEM, FTIR, EDS and XRD. The GO acted as a good supportive substrate for controlling the size and activity of SiO_2_ nanospheres with their cooperation towards catalytic reactions. The SiO_2_ nanospheres with monodispersed sizes are decorated homogeneously on GO nanosheets. Later, this catalytical system used for the synthesis for the synthesis of functionalized quinoxalines by the condensation of OPD with substituted phenacyl bromide/2-diketone at rt in the presence of acetonitrile as a solvent. The phenacyl bromide containing EW and ED groups did not affect significantly on the product yield. The nanostructured SiO_2_-GO hybrid can be easily recovered by a simple filtration method and reused further for a minimum of four cycles without losing considerable activity (Scheme 34).^123^

### 2.6. Titanium dioxide (nano-TiO_2_)-based nanocatalysts for the synthesis of quinoxalines

**Scheme 35:**
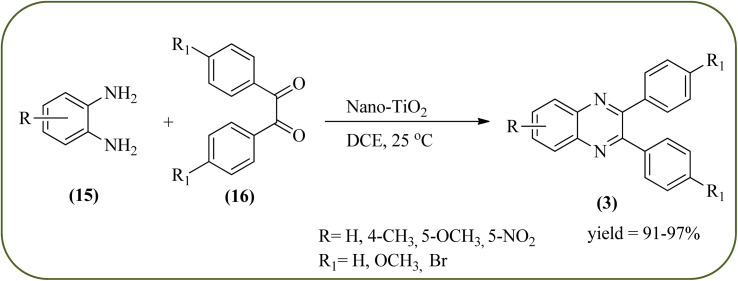
TiO_2_ catalyzed synthesis of quinoxalines.

**Scheme 36:**
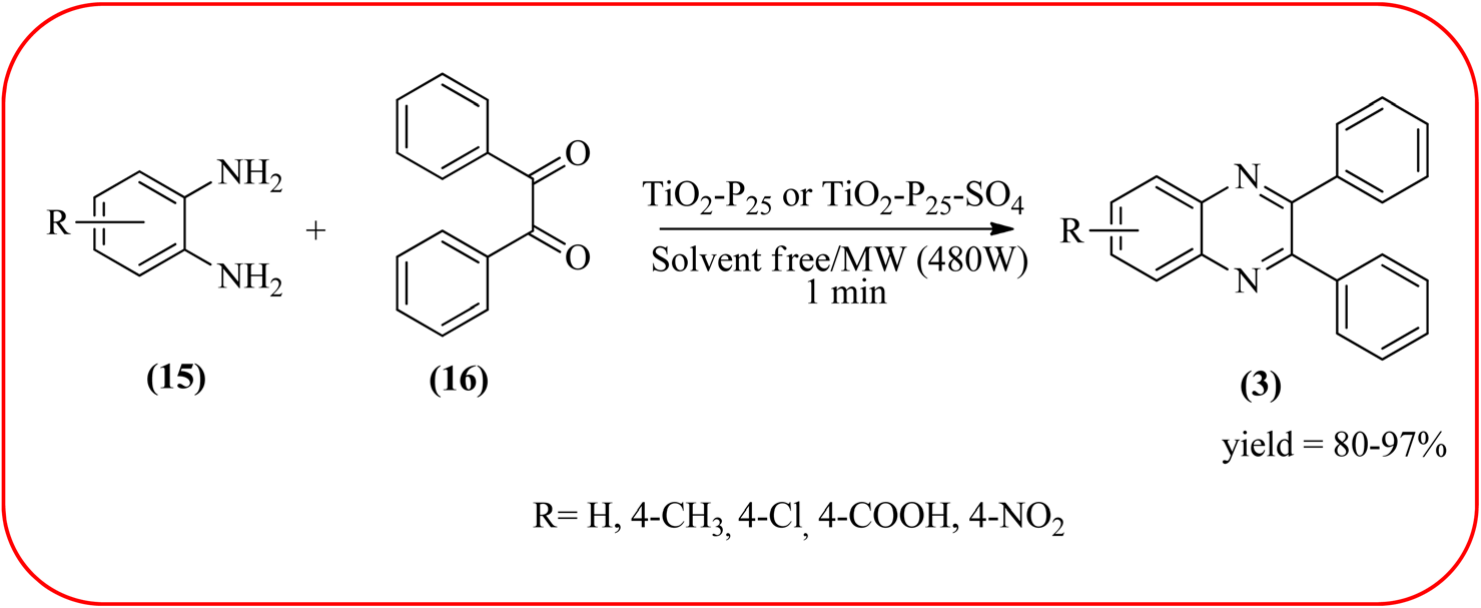
Application of TiO_2_–P25–SO_4_ for the synthesis of quinoxalines.

Titanium dioxide nanoparticles (nano-TiO_2_) are one of the most interesting metal oxides because high activity, strong oxidizing power, easy availability, non-toxicity, reusability, and long-term stability. The nano titanium dioxide is a versatile material for various kinds of industrial applications related to catalysis, photocatalysis for pollutant elimination photovoltaics, or sensors, paints, and organic synthesis.^124,125^ Alinezhad and co-workers synthesized TiO_2_ nanoparticles by the sol–gel method using titanium tetra-isopropoxide, deionized water, ethanol and HNO_3_ under ultrasonic irradiation. The NPs were round in shape, with an average diameter of 50 nm, and further the catalytical activity of nano-TiO_2_ was examined by synthesizing 2,3-diphenyl quinoxaline using various 1,2-diketones with substituted OPDs. The substituted at the 4-position with EDGs in OPD, higher rates, and yields are observed than the ones bearing EWGs at that position. The catalyst was recovered and reused, it shows highly efficient after the fourth run.^126^ Also, Mirjalili *et al.*, have developed the same catalytical system (TiO_2_) for the preparation of quinoxaline derivatives (Scheme 35).^127^ Krishnakumar and Swaminathan have prepared the sulfated TiO_2_–P25 (Degussa titania) (TiO_2_–P25–SO_4_) by sol–gel method using H_2_SO_4_ and utilized this material for the synthesis of quinoxaline, dipyridopyridine derivatives and chalcones under microwave condition. The synthesized material was characterized by FT-IR, XRD, FE-SEM, EDS, HR-TEM, XPS, DRS and BET surface area measurements. These particles exhibit a cloud like structure and size in the range from 20 to 100 nm. The EDGs at the phenyl ring of 1,2-diamine favor the formation of product, while, EWGs such as –fluoro, –chloro, and –carboxy slightly decrease the product yields. The recovered catalyst can be used five times without any loss activity, and it was found to be 98% even at the fifth run (Scheme 36).^128^

### 2.7. Zirconium-based nanocatalysts for the synthesis of quinoxalines

**Scheme 37:**
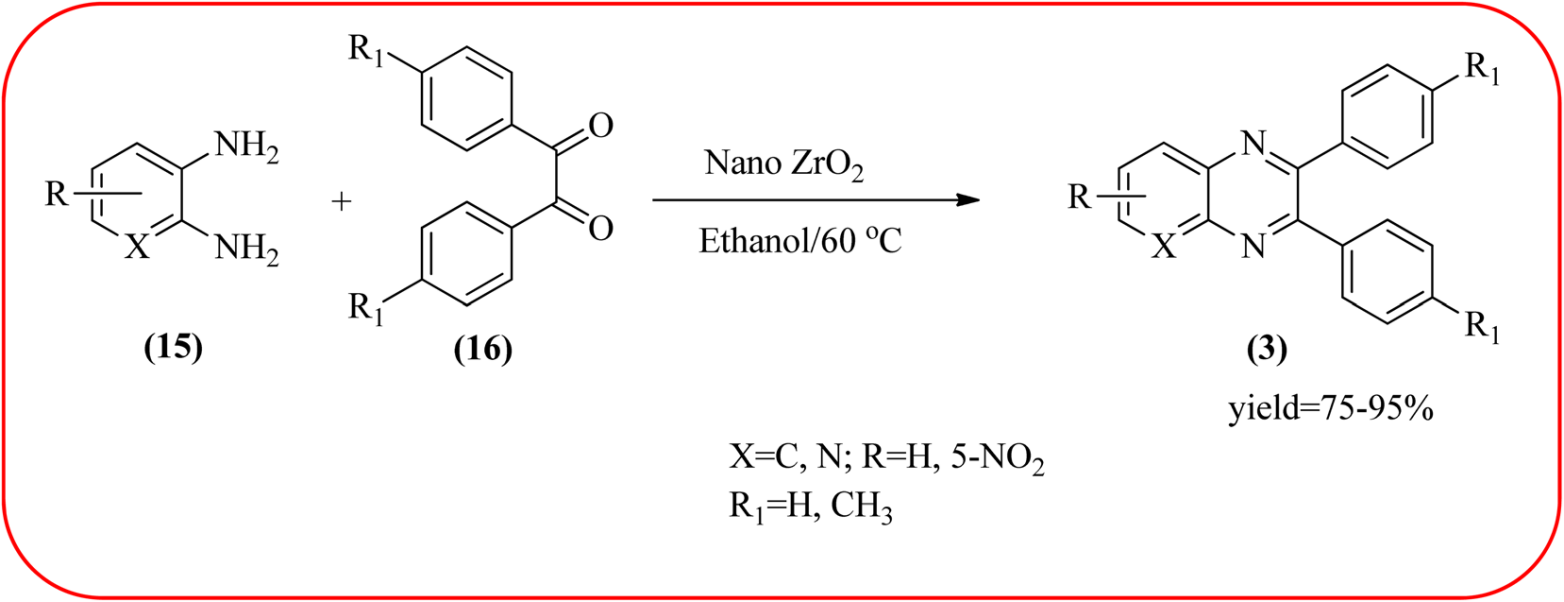
The synthesis of quinoxalines using nano ZrO_2_.

**Scheme 38:**
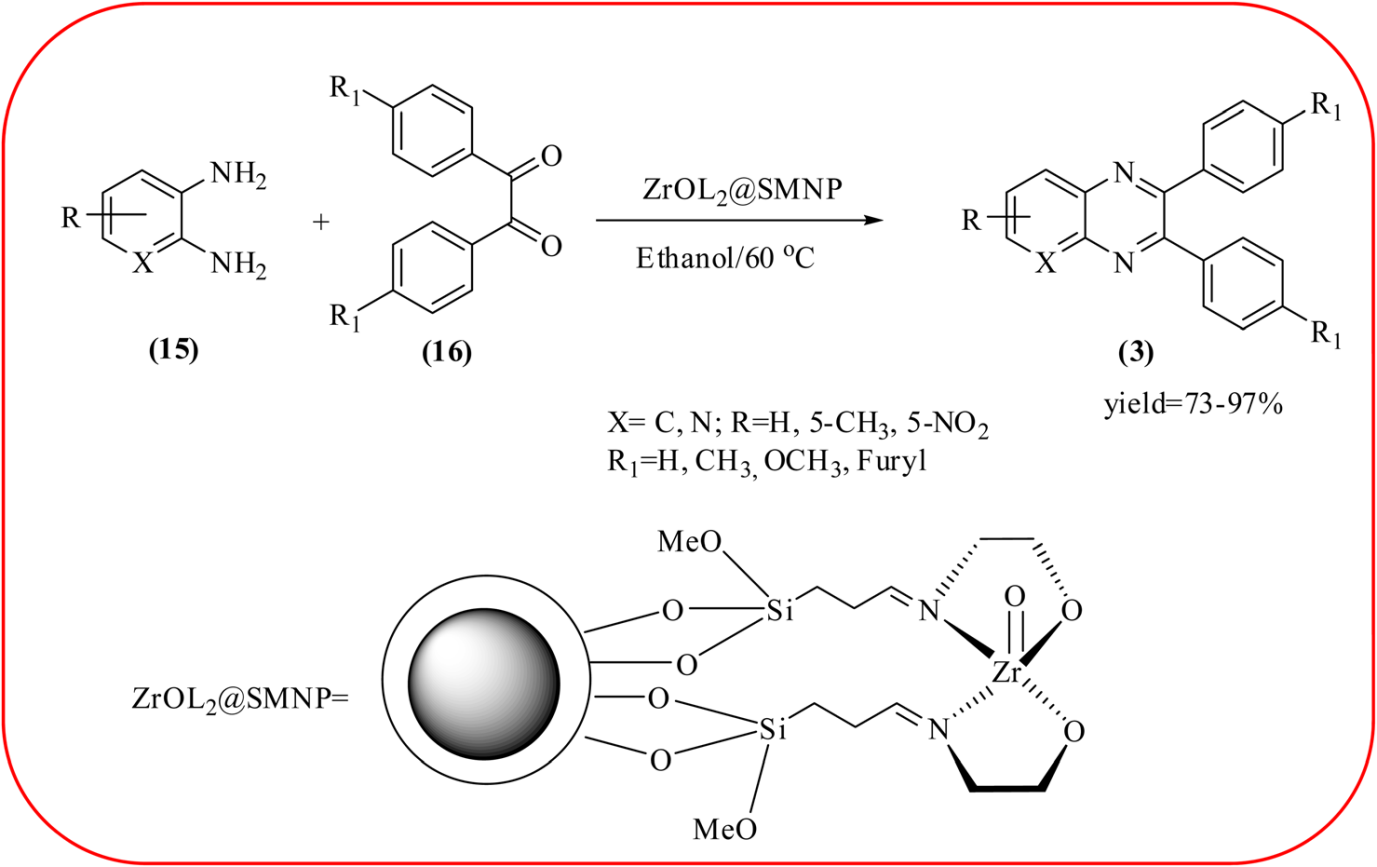
The ZrOL_2_@SMNP catalyzed synthesis of quinoxalines. The black sphere surrounded by white layer represents starch-coated Fe_2_O_3_.

**Scheme 39:**
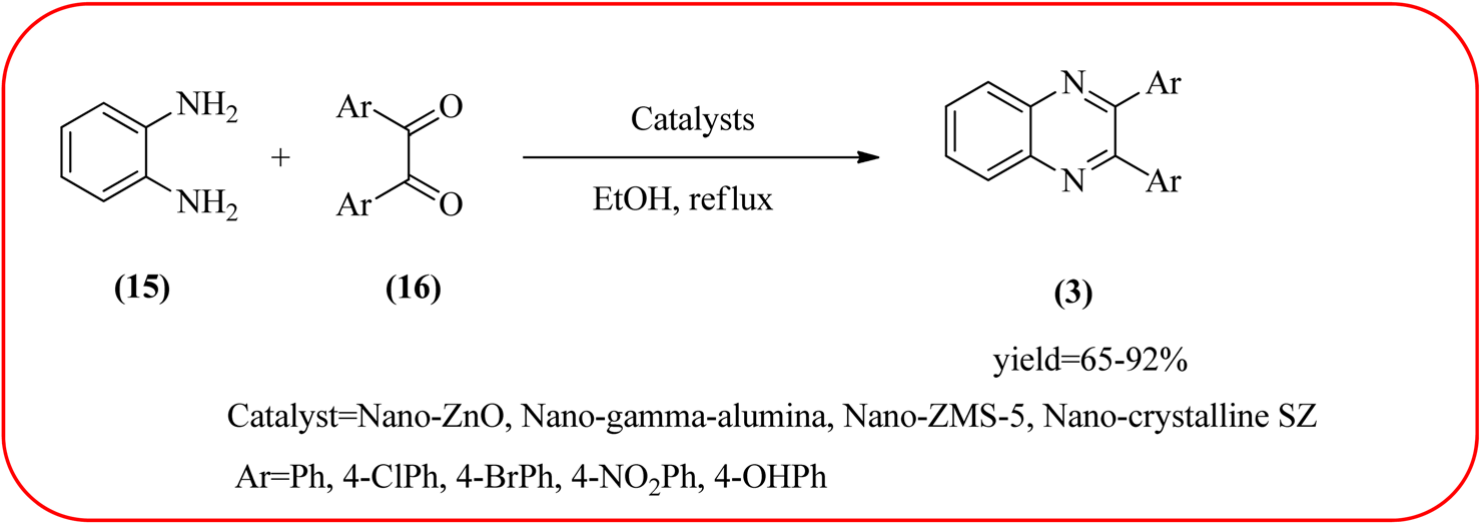
The synthesis of quinoxalines using nanosulfated zirconia, nano-structured ZnO, nano-γ-alumina and nano-ZSM-5 zeolites.

**Scheme 40:**
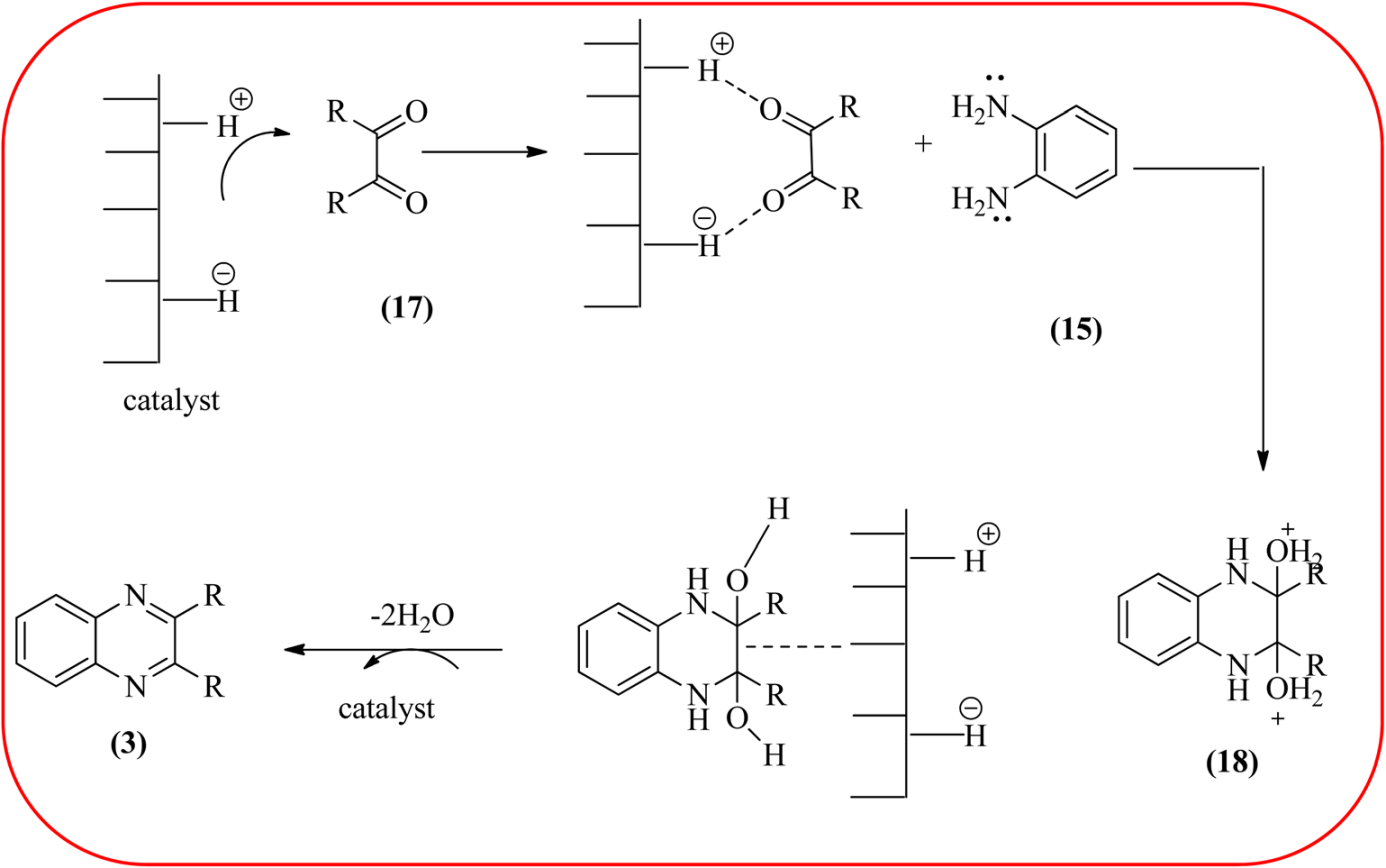
Plausible mechanism for the synthesis of quinoxalines using catalysts (nanosulfated zirconia, nano-structured ZnO, nano-γ-alumina and nano-ZSM-5 zeolites).

In recent years, zirconium-based nanoparticles have attracted much attention from scientists due to their specific optical and electrical properties, and their potential applications in transparent and fuel cells, advanced ceramics, optical devices, catalysis, and sensors.^129,130^ Also, zirconium compounds have found an ever-increasing role in organic synthesis, as revealed by the extensive literature on zirconium compounds as catalysts or reagents in synthetic organic chemistry.^131^ In this section, we were discussed how these materials are used for the synthesis of quinoxalines. Jafarpour and co-workers used monoclinic zirconia nanoparticles as a heterogeneous catalyst for the synthesis of quinoxaline derivatives. This nanomaterial is characterized by spectroscopic methods and reveals the formation of monoclinic zirconia with spherical nanoparticles with 20–40 nm diameter from TEM analysis. Further, the author utilized this material for the preparation of quinoxalines. The condensation reaction of a diamine bearing a strong EWG or a dicarbonyl compound substituted with a strong EDG proceeded slowly. The nano ZrO_2_ can be reusable till the five cycles without the effect of catalytical activity (Scheme 37).^132^ In the same array, the author used zirconium Schiff base complex immobilized on starch-coated maghemite nanoparticles (ZrOL_2_@SMNP). The material is spherical in shape with an average size of 10–14 nm. The catalytical activity is performed by condensation of various 1,2-diamines and 1,2-dicarbonyls for the synthesis of quinoxalines and pyrido pyrazines. The diamine bearing a strongly EWG or a dicarbonyl compound substituted with a strongly EDG proceeded slowly. The catalyst was recovered by decantation of the reaction mixture in the presence of an external magnet and reused it for up to four cycles without loss of catalytical activity (Scheme 38).^133^ Teimouri and co-workers used nano sulfated zirconia, nano-structured ZnO, nano-γ-alumina and nano-ZSM-5 zeolites as the catalyst for the synthesis of different heterocycles like benzimidazoles, benzoxazoles, benzothiazoles, and quinoxalines. Among the four catalytical systems, the nano-sulfated zirconia exhibited greater activity. According to the author, this mechanism involves the coordination of a 1,2-dicarbonyl onto acid sites *via* an acid catalyst, followed by nucleophilic attack on a diketone and dehydration to form a carbocation intermediate and proton elimination to form quinoxalines (Schemes 39 and 40).^134^

### 2.8. Cobalt-based nanocatalysts for the synthesis of quinoxalines

**Scheme 41:**
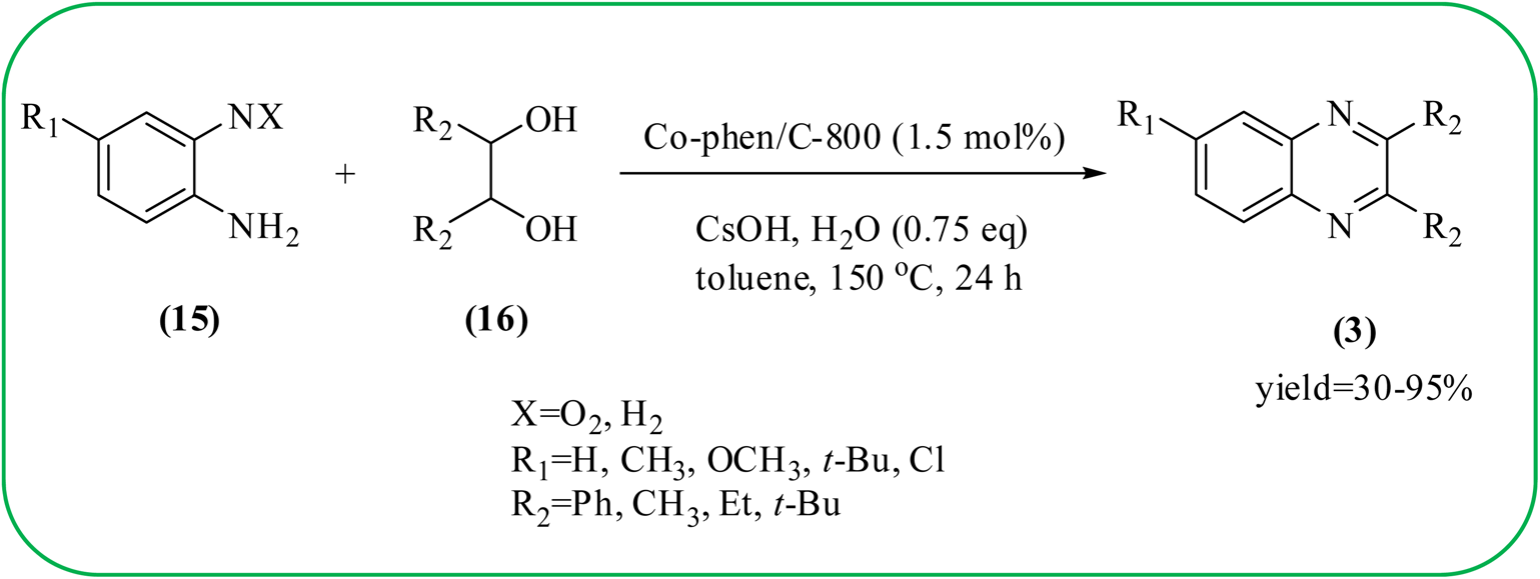
The synthesis of quinoxalines using Co-phen/C-800.

**Scheme 42:**
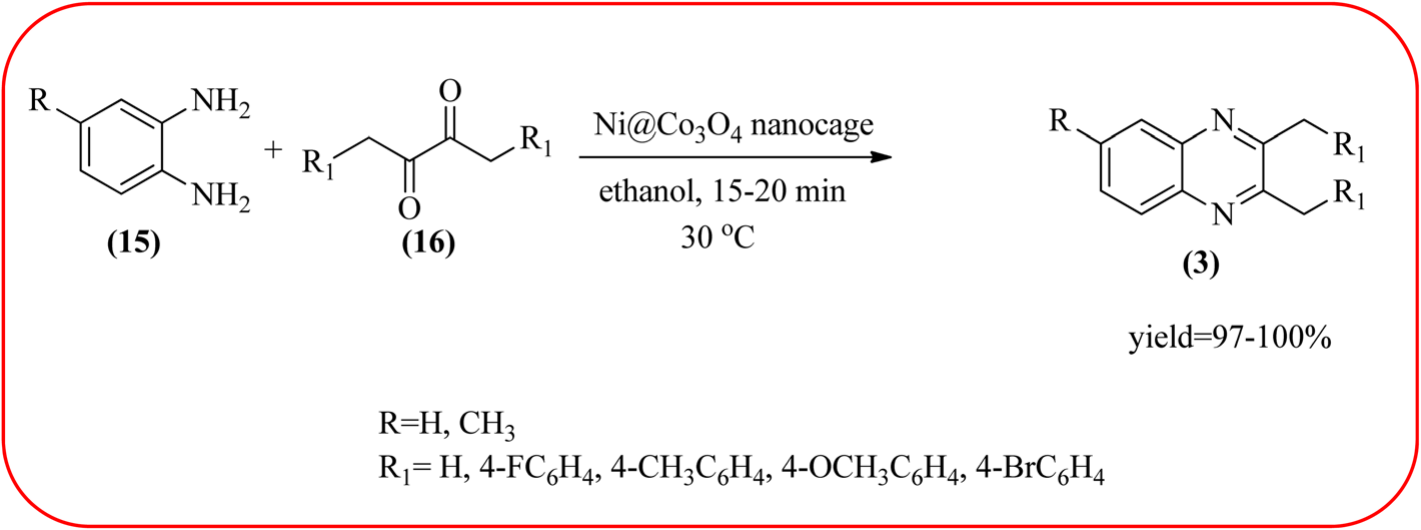
Ni@Co_3_O_4_ (Co_3_O_4_ nanocages decorated with nickel nanoparticles) for the synthesis of quinoxalines.

**Scheme 43:**
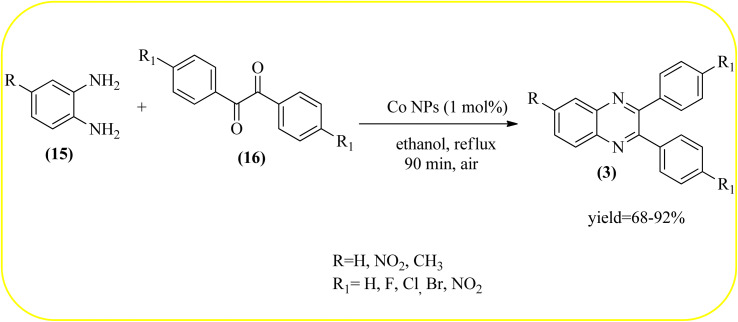
The synthesis of quinoxalines using Co nanoparticle.

Cobalt or cobalt-supported catalysts are an important material system in the field of heterogeneous catalysis. Cobalt as a late 3d transition metal has a rich coordination and organometallic chemistry, which has been explored for synthetic and catalytic purposes.^135,136^ The cobalt NPs (Co-NPs) are used for the synthesis of quinoxalines and are discussed in this section. Nitrogen-doped carbon-supported cobalt nanoparticles from various cobalt precursors and phenanthroline were prepared by Panja and co-workers. In addition to the synthesis of 2-methyl quinoxaline from OPD and dicarbonyl derivatives, cobalt materials were tested for their catalytic activity in coupling nitroarenes with benzyl alcohols to prepare imines. These materials disclosed the existence of CoO and Co_3_O_4_ which were uniformly dispersed over the N-doped carbon support and the average particle size of 17.53 nm and 18.2 nm. Among all the Co-catalysts, the Co-phen/C-800 showed superior results, and the electronic effect with a diol, having diphenyl or *tert*-butyl substitution lowers the yield of the desired product. The catalyst was recycled and used up to 8 cycles and this heterogeneous system did not lose its catalytic activity significantly (Scheme 41).^137^ Sharma and co-workers synthesized Co_3_O_4_ nanocages decorated with nickel nanoparticles Ni@Co_3_O_4,_ an average size of about 65 nm. These cage materials have hollow architectures that can help in achieving high reactivity and intensifying their role in various important organic reactions. The catalytical system was applied for the synthesis of quinoxalines with numerous 1,2-diketones and 1,2-diamines. Substrates containing deactivating groups such as –Cl, –Br, *etc.* required a longer reaction time. Also, this methodology is useful with aliphatic 1,2-diketones, resulting in a good conversion percentage and exhibiting a high turnover number. The nanocomposite Ni@Co_3_O_4_ was reprocessed/recycled by simple centrifugation and used up to six times without any appreciable loss in its catalytic activity (Scheme 42).^138^ The cobalt nanoparticles on mesoporous SBA-15 were synthesized by Rajabi and co-workers. Later this catalyst was applied for the preparation of quinoxalines from 1,2-diamines and 1,2-dicarbonyls under mild reaction conditions. The supported nanocatalyst exhibited excellent activity, stability and it could be reused at least ten times without any loss of catalytical activity (Scheme 43).^139^

### 2.9. Molybdenum-based nanocatalysts for the synthesis of quinoxalines

**Scheme 44:**
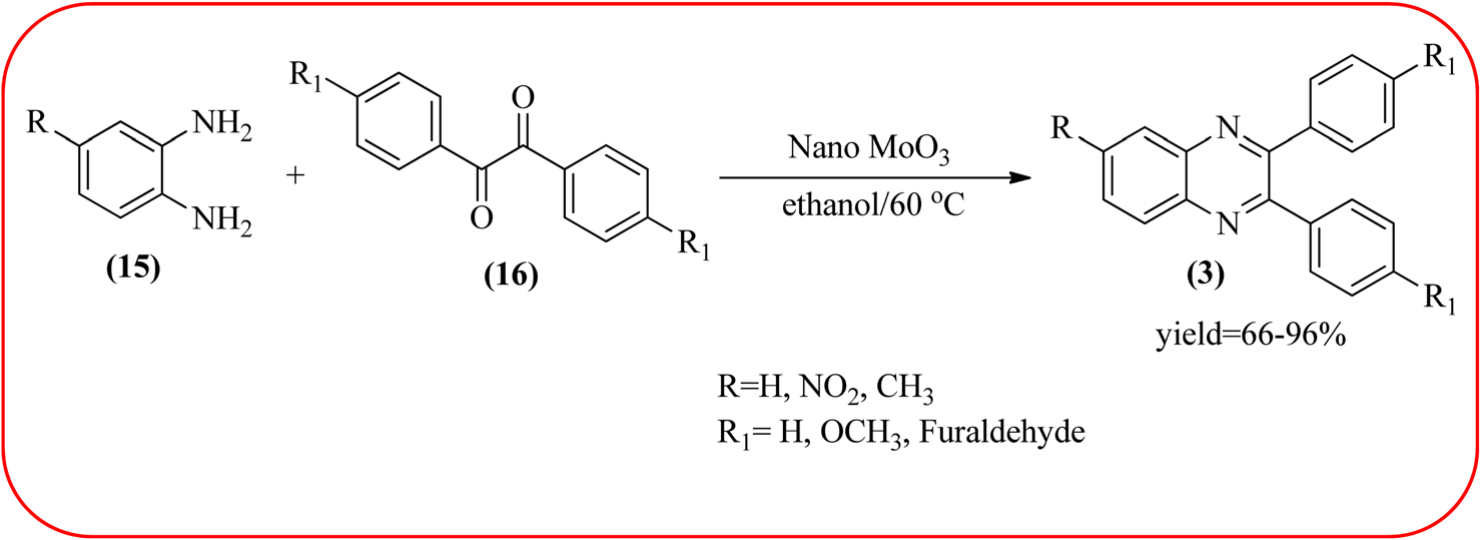
Nano-MoO_3_ catalysed synthesis of quinoxalines.

**Scheme 45:**
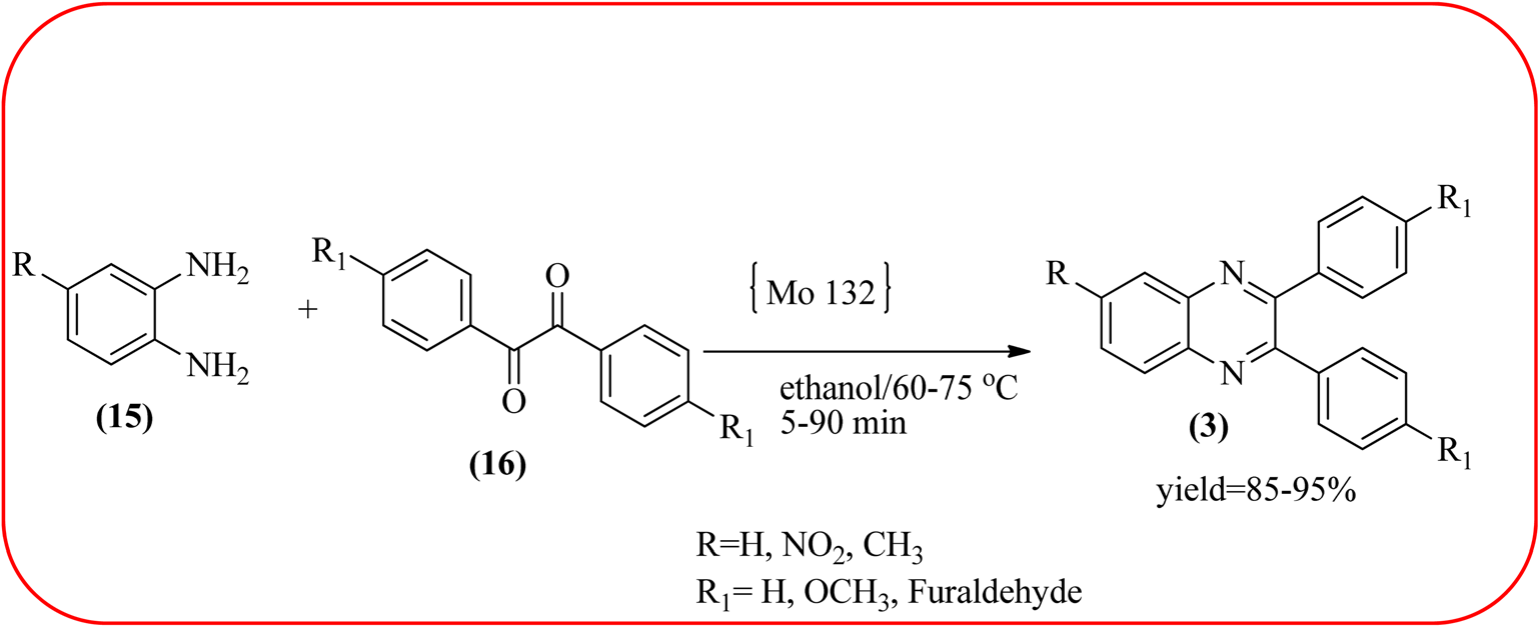
Synthesis of quinoxalines using keplerate {Mo132} nanoball.

**Scheme 46:**
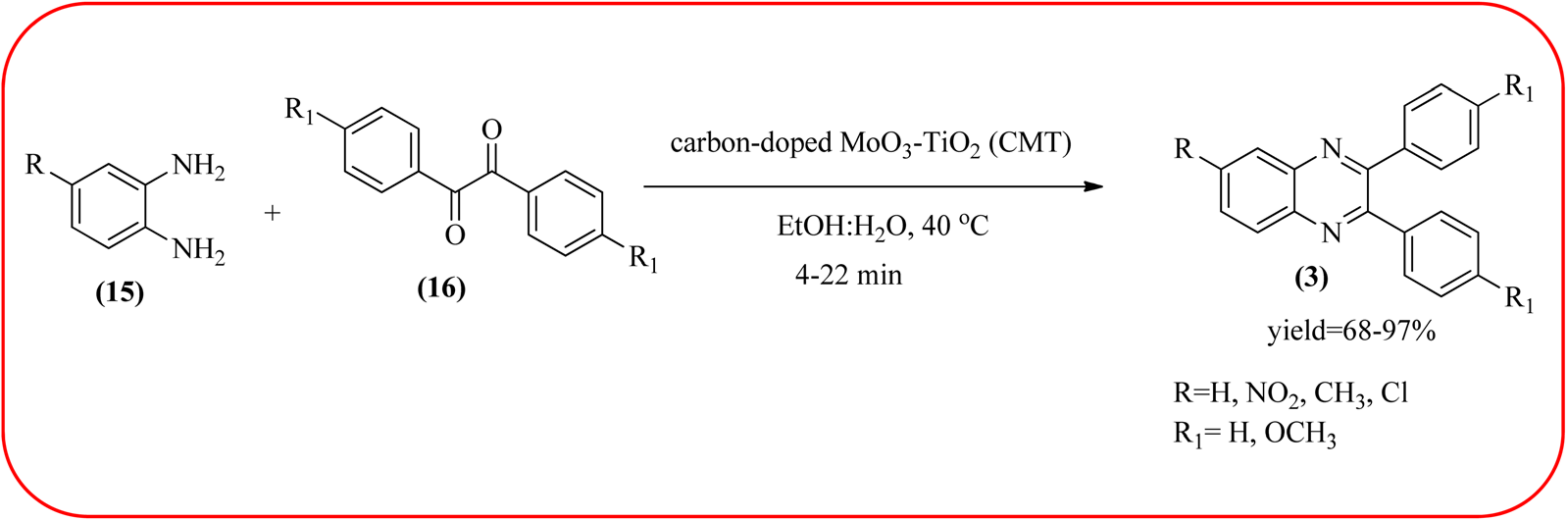
Application carbon-doped MoO_3_–TiO_2_ (CMT) for the synthesis of quinoxalines.

The molybdenum-based nanomaterial/nanocatalysts are extremely important for industrial applications. Mo-based catalysts can be active and selective for many reactions. Hydrogenation of benzene, selective oxidation hydrogenation/dehydrogenation, and cracking of hydrocarbons, are a few examples of the important applications of these catalysts.^140,141^ The Mo-based nanocatalysts were used for the synthesis of nitrogen-containing heterocycles like quinoxaline and the same was discussed in this section. The crystalline nanobelts of α-MoO_3_ nanomaterial were prepared by Jafarpour and co-workers. The material was characterized by XRD, FT-IR, Raman spectroscopy, HRTEM, SEM, and TPD (temperature-programmed desorption). From TEM analysis, it is confirmed, the nanobelt morphology of α-MoO_3_ ranged from 20–70 nm in width and 200–400 nm in length. Further, these α-MoO_3_ nanobelts were utilized as a heterogeneous catalyst for the synthesizing quinoxaline derivatives. The condensation reaction of a diamine bearing a strong EWG or a dicarbonyl compound substituted with a strong EDG proceeded slowly. The catalyst can be recovered after the reaction; it is separated by centrifuging followed by decantation. The isolated solid phase (nano-MoO_3_) was then dried under reduced pressure and reused for five runs without any noticeable drop in the product yield and its catalytic activity. The main advantages of this methodology are environmentally friendly, cost-effective, and industrially important because of the catalyst reuse and the use of safe reaction media (Scheme 44).^142^ In the same array, the author used keplerate {Mo132} nanoball for the synthesis of quinoxaline derivatives. These clusters showed ball morphology with sizes ranging between 5 and 25 nm. The condensation reaction of a diamine with a strong EWG, and a dicarbonyl compound substituted with a strong EDG, did not proceed quickly. The author proposed the plausible mechanism, the main role of the {Mo132} acts as a Lewis acidity of the plenty of Mo centers in a highly ordered structure which leads to their effective interactions with the carbonyl oxygen atoms of 1,2-diketone, thereby increasing the polarization of carbonyl moiety and promoting the cyclo-condensation reaction (Scheme 45).^143^ Lande and co-workers prepared carbon-doped MoO_3_–TiO_2_ (CMT) material by sol–gel method. For the carbon source, the natural wood plant Acacia Arabica was used. Further, this material was characterized by XRD, SEM, EDS, and FT-IR. The material is with agglomeration and randomness in the particle size with 10–15 nm. Later, this material was applied for the preparation of quinoxaline derivatives from benzil and *ortho*-1,2-diamine in EtOH : H_2_O (3 : 1) solvent system at 40 °C by using the ultrasonication method. The catalyst can be recovered and reused at least three times without significant loss in catalytic activity. The merits of this method are mild reaction conditions, short reaction time, high yield, simple experimental procedure, efficient, environmentally benign, and green synthetic protocol (Scheme 46).^144^

### 2.10. Manganese-based nanocatalysts for the synthesis of quinoxalines

**Scheme 47:**
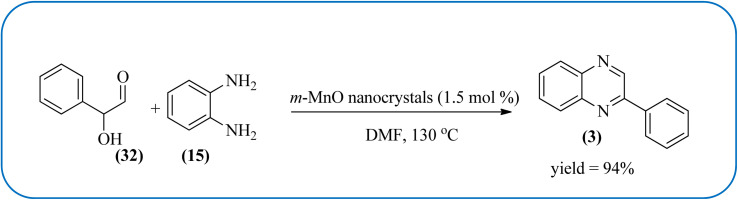
Synthesis of quinoxalines using manganese oxide (MnO) nanocrystals.

**Scheme 48:**
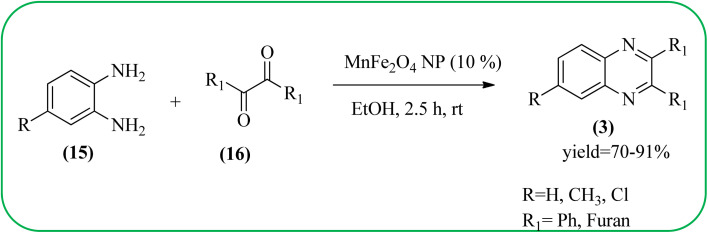
MnFe_2_O_4_ catalyzed synthesis of quinoxalines.

Manganese-based nanomaterial has attracted scientists due to its wide range of applications like MRI contrast agents, catalysts, electrode materials, and biomedical materials owing to their unique chemical, physical, and magnetic properties.^145,146^ These materials are used as a heterogeneous catalyst for the synthesis of heterocycles like quinoxalines and the same is discussed in this section. Using an Mn-oleate complex, Kim *et al.*, prepared manganese oxide (MnO) nanocrystals that are single crystalline and uniformly multimodal, with an average distance of 47 nm between the tips. Further, these nanocrystals were utilized as a catalyst for the synthesis of a quinoxaline from an α-hydroxy ketone. The author did the optimization of solvent (DMF, methanol, DCM), temperature, catalyst concentration, and reaction time. The reaction was performed in the presence of DMF at 1.5 mol% *m*-MnO NCs at a temperature of 130 °C for 6 h. Through this method, the reaction rate was remarkably enhanced, and very high conversion was observed. Also, *m*-MnO NCs and *s*-MnO NCs exhibit different activities depending on their shape. The author demonstrated, this by comparing the activities of the *m*-MnO NCs and the *s*-MnO NCs. The spherical MnO nanoparticles were used as catalysts in the synthesis of a quinoxaline derivative. The reaction performed with the use of the *s*-MnO NCs as a catalyst resulted in a low conversion of 60% under the same conditions that provided a higher conversion with the *m*-MnO NCs. The catalyst can regenerate and be reused three times with a slight loss in catalytic activity (Scheme 47).^147^ Brahmachari and co-workers used magnetically separable MnFe_2_O_4_ material as a heterogeneous catalyst for the synthesis of quinoxalines and 2-substituted benzimidazoles at rt under aerobic conditions. The average size of the nanocrystals was in the range of 50–100 nm. Manganese ferrite (MnFe_2_O_4_) is one of the most important spinel ferrite magnetic oxides where oxygen has fcc close packing and Mn^2+^ and Fe^3+^ ions can occupy either tetrahedral or octahedral interstitial sites. The major advantages of this protocol are easy separation of the heterogeneous catalyst from the reaction mixture, its reusability, high selectivity, room temperature condition, energy efficiency, operational simplicity, clean reaction profiles, and good yields (Scheme 48).^148^

### 2.11. Ruthenium- and zinc-based nanocatalysts for the synthesis of quinoxalines

**Scheme 49:**
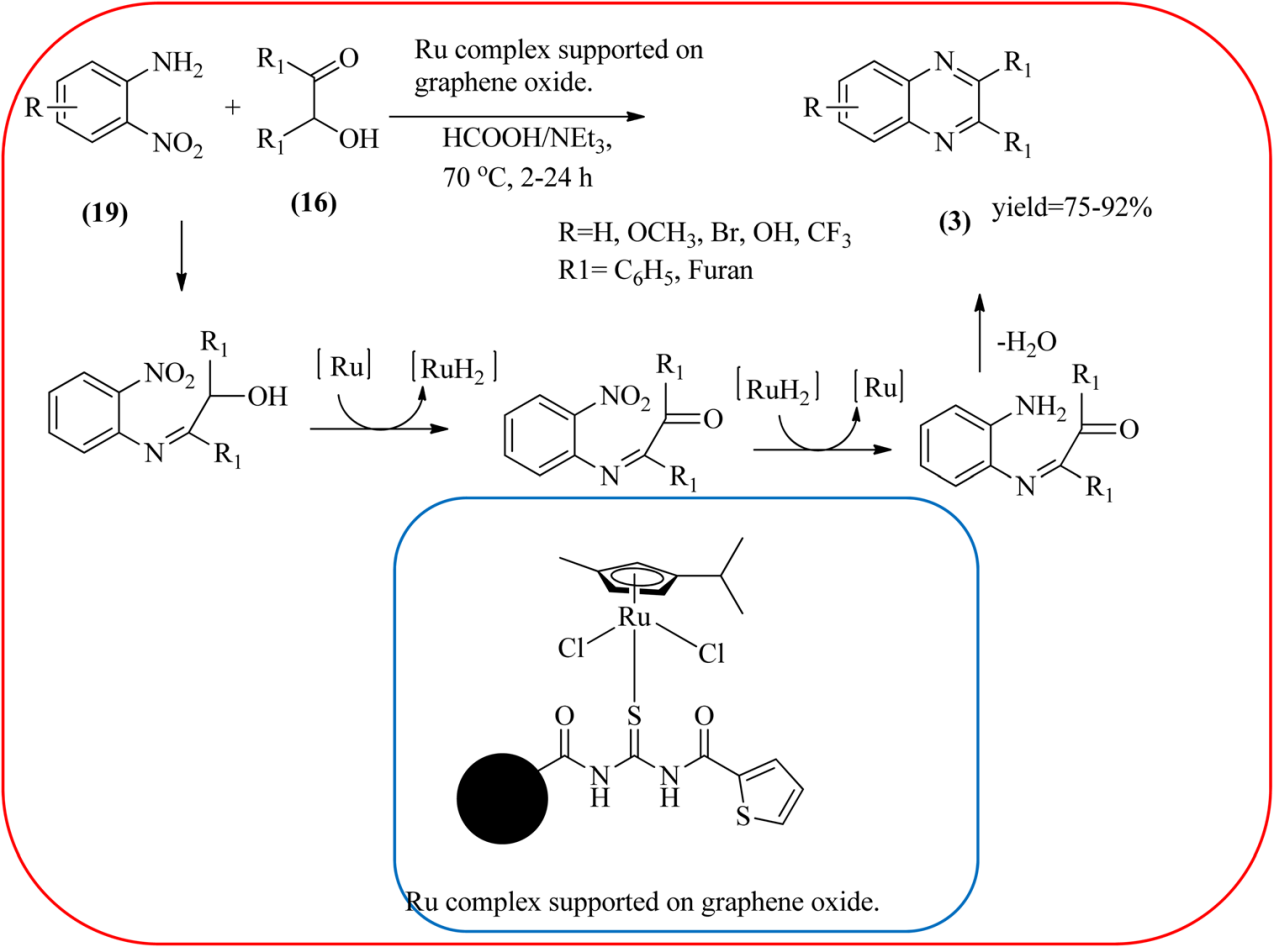
Synthesis of quinoxalines using Ru complex supported on graphene oxide.

**Scheme 50:**
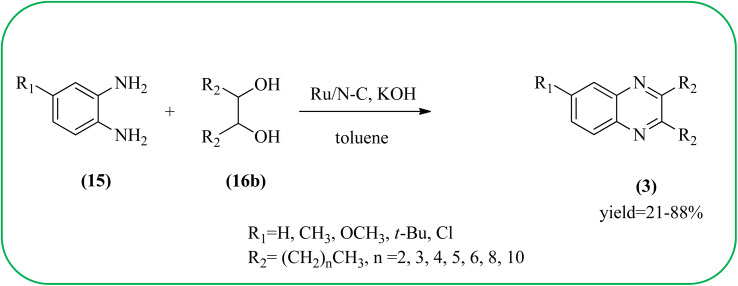
Application of Ru NPs for the synthesis of quinoxalines.

Sindhuja and co-workers reported Ru complex supported on graphene oxide as a catalyst. The catalyst was used for the synthesis of quinoxaline derivatives from various 2-nitroaniline and hydroxy ketone derivatives *via* the transfer hydrogenation approach. The Ru(ii) complexes are homogeneous catalysts that have been heterogenized by supporting it on graphene oxide. The nitroaniline substrates were smoothly combined with the dicarbonyl compound in the presence of a catalyst, and the products were obtained in 65–81% yields. They catalyzed the conversion of 2-nitroaniline and benzoin into 2,3-diphenyl quinoxaline in 8 h with a yield of 83%. The 6-methoxy-substituted quinoxaline and 6,7-dimethyl-substituted quinoxaline were obtained in 66 and 68% yields. In the nitroaniline, with a methyl group, the reaction did not complete even after 24 h, and the maximum conversion was 54% with 100% selectivity. The author predicted the plausible pathway for quinoxaline formation, in the first step, there was no formation of diamine from 2-nitroaniline, which confirmed that the first step was imine formation, followed by transfer hydrogenation and cyclization (Scheme 49).^149^ Guo *et al.* have prepared Ru NPs on N-doped carbon support and utilized the material for the synthesis of benzimidazoles and quinoxaline acceptor less dehydrogenation coupling (ADC) reaction of diamines with primary alcohols or diols. The Ru/N–C catalyst can be recycled and used up to five times without significant loss of activity. Alkylethane-1,2-diols like propane-1,2-diol, butane-1,2-diol, pentane-1,2-diol, hexane-1,2-diol, octane-1,2-diol, and dodecane-1,2-diol and butane-2,3-diol required higher a diol/molar ratio and a higher temperature (130 °C) to complete the reactions (Scheme 50).^150^

**Scheme 51:**
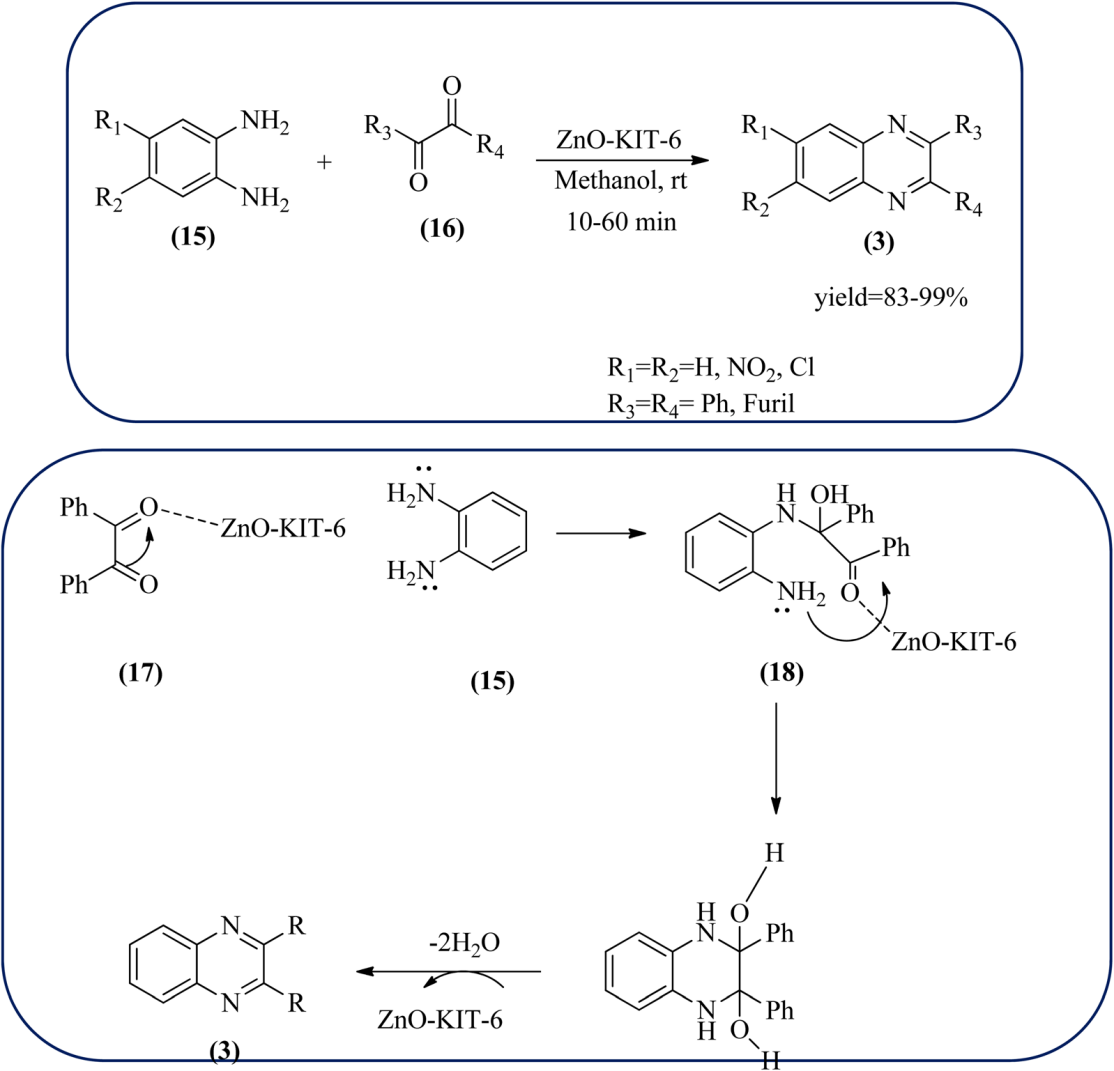
ZnO-loaded mesoporous silica (KIT-6) as an efficient solid catalyst for synthesis of quinoxalines and its plausible mechanism.

Zinc oxide (ZnO) is well known to be highly active, recyclable, non-toxic, and abundantly available for several organic transformations. In this context, Hamid *et al.*, have used ZnO nanoparticle-loaded highly ordered mesoporous silica KIT-6 materials as solid catalysts as an efficient catalyst for the synthesis of various substituted quinoxalines using OPDs and diverse ketones as the reactants at rt in methanol as the solvent. These ZnO nanoparticles with an average particle size of around 9 nm were indeed formed inside the KIT-6-T mesopores with the wet impregnation method. The reaction works out very well with EDG and ERG in OPD (–chloro, –nitro, –dichloro, –cyclohexyl, and bis-OPD) and also various aromatic diketones such as benzyl and furyl which were used to produce various kinds of quinoxaline. The author proposed the plausible mechanism, the catalyst acts as Lewis acid and activates the di-ketone, followed by condensation, to yield the final product (Scheme 51).^151^

### 2.12. Palladium- and phosphotungstic acid-based nanocatalysts for the synthesis of quinoxalines

**Scheme 52:**
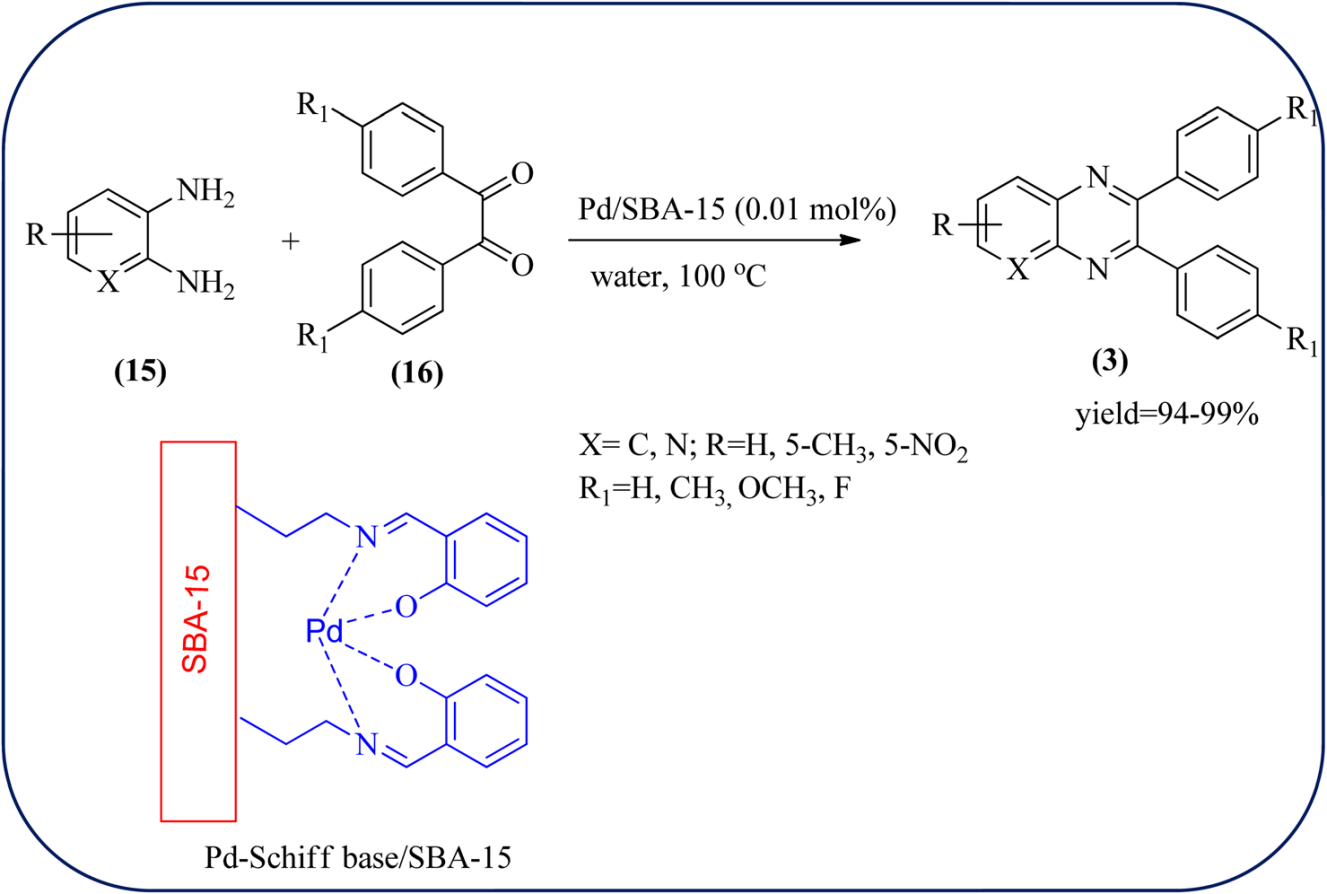
Synthesis of quinoxaline derivatives catalyzed by Pd/SBA-15.

**Scheme 53:**
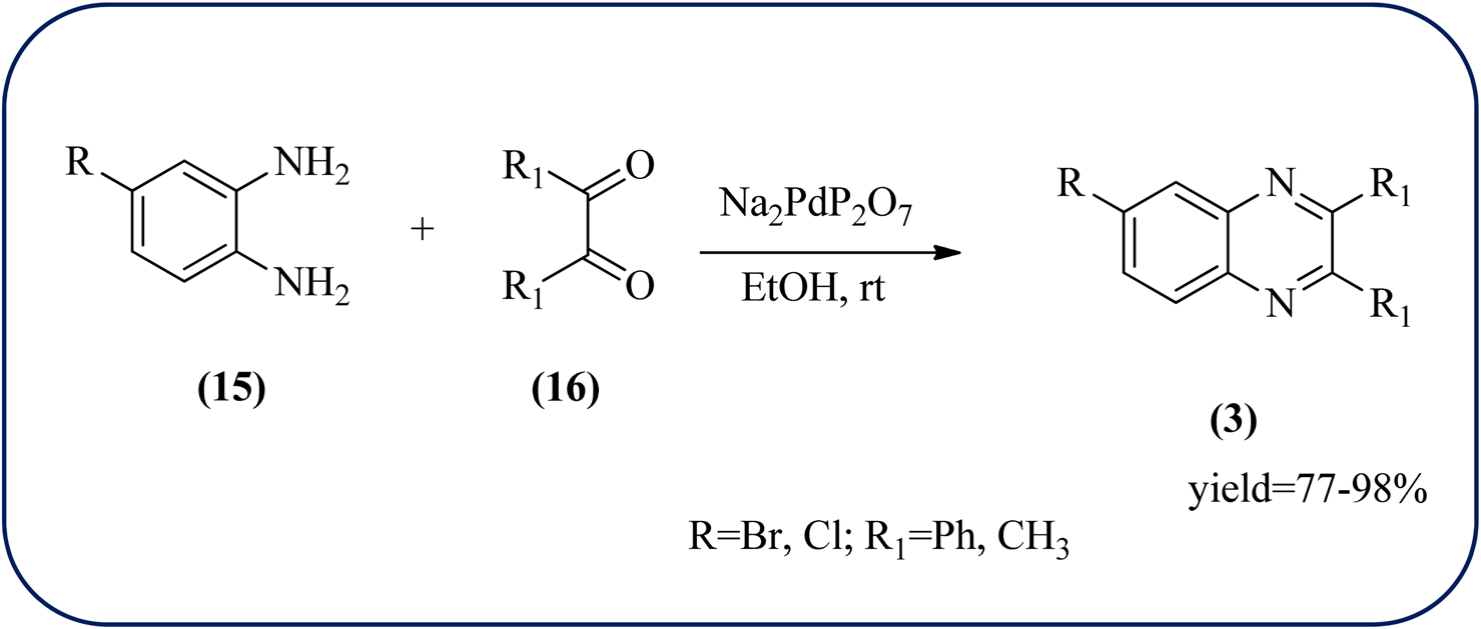
Na_2_PdP_2_O_7_ as an efficient catalyst for synthesis of quinoxalines and its plausible mechanism.

Palladium (Pd) and Pd-based nanomaterial has become an area of intense interest. Pd is well-known for its high affinity for hydrogen, which facilitates the broad use of Pd nanomaterials as primary catalysts, encompassing a wide variety of applications, particularly in organic coupling synthesis, hydrogen detection, purification, and storage.^152^ Bardajee and co-workers prepared an SBA-15-supported palladium catalyst containing N–O chelating Schiff-base ligand from SBA-15 and a Pd(ii) Schiff-base complex. Further, these materials were characterized by IR, XRD, nitrogen adsorption–desorption method, TEM, and BET analyses. From XRD analysis, the strong peak corresponding to (100) plane and two weak peaks corresponding to (110) and (200) planes of ordered hexagonal mesoporous materials, and TEM analysis reveals Pd(ii)-Schiff base/SBA-15 are the ordered channel structure of mesoporous materials which is retained during the complex grafting. The BET analysis showed material exhibited slightly smaller pore volumes, interplanar spacing, and a slightly larger wall thickness. The catalyst/material was applied for the synthesis of quinoxaline, [1,2-b]pyrazine, pyrido[2,3-*b*]pyrazine, and pyrido[3,4-*b*]pyrazine derivatives from 1,2-diamines and 1,2-diketones. In general, Pd/SBA-15 catalyzed reactions produced good to excellent conversions without generating undesirable side products. The OPD-bearing EWG and diamino pyridines were more slowly condensed and needed longer reaction times and electron-rich aromatic diamines gave preferred products in shorter reaction times. Also, Pd/SBA-15 showed excellent reusability over eight successive runs under similar conditions (Scheme 52).^153^ Dânoun *et al.*, prepared nanostructured pyrophosphate Na_2_PdP_2_O_7_ bifunctional heterogeneous catalyst. The material with average crystallite size was around 7.9 nm with agglomerates in shape. The material was used for the synthesis of quinoxaline derivatives to give a good yield. The variety of substrates used and the presence of EDG such as methyl group on benzene-1,2-diamine substrate did not affect the reaction time and yield. On the other hand, EWG in benzene-1,2-diamine decreased the rate of reaction and yields when compared to unsubstituted substrate. The catalyst can be easily separated and reused in several cycles with a slight drop in the catalytical activity. The main features of the protocols are easy work-up, good yield, short reaction time, and eco-friendly process (Scheme 53).^154^

**Scheme 54:**
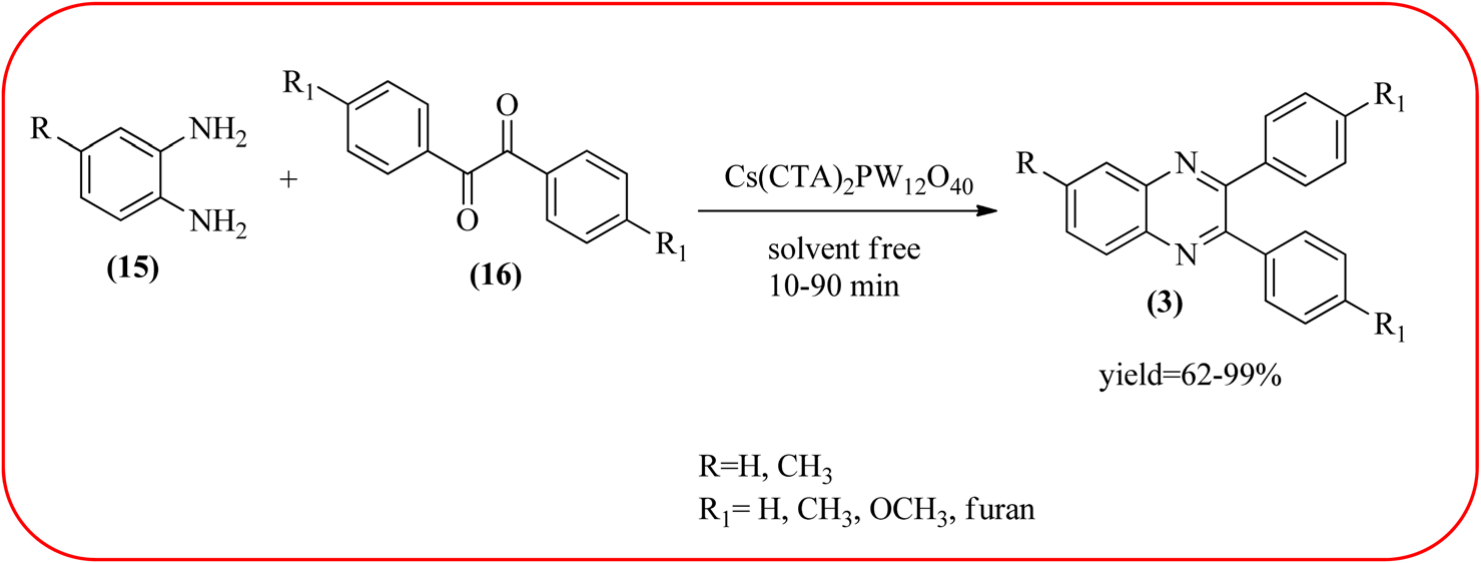
Synthesis of quinoxalines using Cs(CTA)_2_ PW_12_O_40_ as a catalyst.

**Scheme 55:**
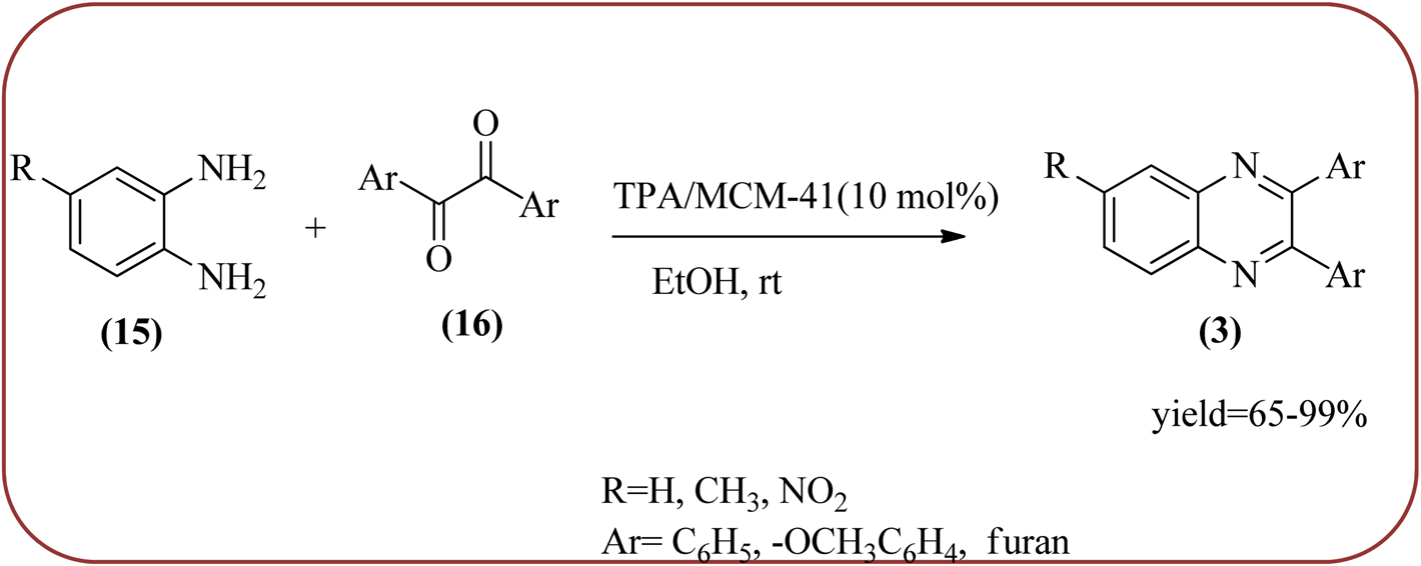
Synthesis of quinoxalines in the presence of 10 wt% TPA/MCM-41 at room temperature in EtOH.

Phosphotungstic acid (PTA) or 12-tungstophosphoric acid (TPA), as a Keggin-type heteropoly acid, has attracted considerable attention as an acid catalyst in organic transformations because of its high Brønsted acidity, and thermal stability.^155^ Masteri-Farahani and co-workers have prepared Keggin-type Cs(CTA)_2_PW_12_O_40_ (CTA = cetyltrimethylammonium cation) nanostructure from aqueous solution of phosphotungstic acid to the microemulsion solution. These materials are star-shaped nanostructures composed of some nanorods with a diameter and lengths of about 100 nm and 500 nm respectively. Further, this material was utilized for the preparation of quinoxaline derivatives from the various 1,2-dicarbonyl and 1,2-diamine under solvent-free conditions. All the reactions proceeded very cleanly giving the desired product without any side product. The catalyst was regenerated by filtration and reused in the fresh reaction mixture with no significant change in product yield, indicating that the nanostructure was stable during the catalytic cycle. The present methodology has several advantages such as high yields, cleaner reactions, short reaction times, and minimal environmental impact, which make it a useful process for the synthesis of quinoxaline derivatives (Scheme 54).^156^ Abdollahi-Alibeik *et al.* have synthesized nano-sized mesoporous silica (MCM-41) supported 12-tungstophosphoric acid (TPA) as a solid acid catalyst and characterized by XRD, FT-IR, and SEM techniques. The material is agglomerated nanoparticles with a size range of less than 100 nm. Further, this catalyst was applied for the synthesis of quinoxalines by the reaction of OPD and 1,2-diketones under solvent-free conditions. The author studied, the effect of the loading amount of TPA on the catalytic activity of TPA/MCM-41 ranging from 5–15 wt% TPA, and the best results were shown at 10 wt% TPA/MCM-41. The various quinoxalines were prepared with EDG and EWG on the OPD and 1,2-diketones, obtained in high to excellent yields. The catalyst TPA/MCM-41 was recovered from the reaction, washed with EtOH, and dried. Further, it can be used for three more cycles without appreciable change in its activity and shows only a slight decrease in the yield after the first run (Scheme 55).^157^

## 3 Conclusion

The quinoxaline ring is an important pharmacophore in modern drug discovery. Therefore, a wide variety of bioactive quinoxalines has been realized as anticancer, antibacterial, anti-inflammatory, antimicrobial, and antiviral agents. Hence, syntheses of this core have become a hot topic for researchers worldwide. Researchers are developing several greener conditions for the synthesis quinoxalines over the last 2–3 decades, especially using the nanocatalyst. This review comprehensively summarizes the role of nanocatalysts for synthesizing pharmacologically important quinoxaline using *o*-phenylenediamine condensed with various diketone. The catalytic protocols utilizing nanocatalysts and their plausible reaction mechanisms have been discussed throughout the review (Table 1). These nanocatalysts/nanomaterials acts as heterogeneous catalysts were found to be heterogeneous, reusable, economically viable and having an environment-friendly nature. The main advantages of these catalysts were found to render quinoxaline in good yields with easy workup, short reaction time, mild conditions, minimal waste, low cost, atom efficiency, safety, solvent-free (some methods), and possessing excellent functional group tolerance. All the methods followed the same mechanistic pathways revealed condensation, cyclization followed by the use of oxidant or removal of water. However, developing novel methodologies using more efficient catalytic systems to synthesize a variety of biologically active quinoxaline scaffolds with better stereoselectivity, regioselectivity, and chemo selectivity is always in need. The results described in this review will encourage synthetic chemists to develop more convenient and eco-friendly novel catalytic systems with a high atom economy in the future.

**Table 1:** Synthesis of quinoxaline using *o*-phenylenediamine with various diketone using different catalysts at various reaction conditions

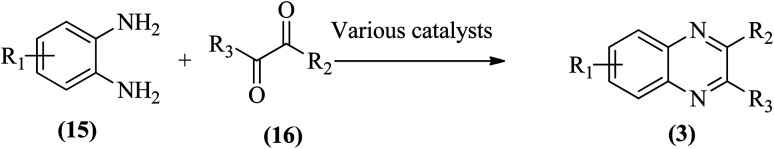				
S. no	Catalyst	Reaction conditions	Yield (%)	Reference
1	Fe_3_O_4_@SiO_2_/Schiff base complex of metal ions	EtOH/H_2_O, rt, 10–120 min	87–97%	88
2	Fe_3_O_4_ nanoparticles	H_2_O, rt, 1–5 h	86–95%	89
3	Fe_3_O_4_@APTES@isatin-SO_3_H	EtOH/rt, 20–70 min	85–95%	90
4	Fe_3_O_4_@APTES@MOF-199	EtOH/rt, 10–40 min	85–97%	91
5	Silicon carbonitride-based iron (Fe@SiCN)	6.5 MPa H_2_, 125 °C., 24 h, H_2_O/TEA	86–95%	92
6	Fe(iii)-Schiff base/SBA-15	H_2_O, 2 h	95–99	93
7	Fe_3_O_4_@SiO_2_@5-SA MNPs	H_2_O, 60 °C, 40–150 min	70–95%	94
8	Fe_3_O_4_@SiO_2_-imidazole-PMA magnetic porous nanosphere	EtOH/rt, 7–25 min	89–95%	95
9	Nano-kaoline/BF_3_/Fe_3_O_4_	Grinding, 10–75 min	74–98%	96
10	Nano-γ-Fe_2_O_3_–SO_3_H	Solvent-free, 120 °C, 1 h	76–98%	97
11	PBS@SMAP	EtOH, 50 °C, 30 min	81–96%	98
12	5% Fe/ZnO catalyst	MeOH, rt, 15–60 min	83–95%	99
13	Leave extract of *Boswellia serrata* plant and the FeNPs	H_2_O, rt, 7–25 min	85–98%	100
14	Cu(ii)-Schiff base/SBA-15	H_2_O, rt, 120 min	93–99%	104
15	Cu(ii)-DiAmSar/SBA-15	H_2_O, rt, 5–30 min	93–99%	105
16	Cu–Al-2	K_2_CO_3_, DMAP, 70 °C, 10 h	55–99%	106
17	CuO	H_2_O, rt, 30–40 min/ultra-sonication	84–98%	107
18	g-C_3_N_4_/Cu_3_TiO_4_ (CNCT) nanocomposite	EtOH, *hv*, sonication, 3–5 min	83–98%	108
19	Ni(ii) ion-loaded Y-type zeolite (NNZ) material	EtOH, rt, 5–50 min	75–92%	111
20	Ni-nanoparticles (14–18 nm)	Acetonitrile, 25 °C, N_2_ atmosphere	83–98%	112
21	Au/CeO_2_	Diglymine, 140 °C, 24 h	73–91%	114 and 115
22	Au-NPs	K_2_CO_3_, H_2_O, air, 80 °C, 2–5 h	82–93%	116
23	SiO_2_ nanoparticles	Solvent-free, rt, 5–25 min	76–92%	119
24	37% Nano-BF_3_.SiO_2_	Solvent-free, rt or sonication	82–96%	120
25	Tungstophosphoric acid/mesoporous silicas (SNPX#WPA)	Toluene, 20 °C	75–98%	121 and 122
26	Silica-based nanosphere-graphene oxide (SiO_2_-GO) hybrid	CH_3_CN, rt	89–90%	123
27	Nano-TiO_2_	DCE, 25 °C, 15–60 min	91–97%	126 and 127
28	TiO_2_–P25 or TiO_2_–P25–SO_4_	Solvent free/MW (480 W), 1 min	80–97%	128
29	Nano ZrO_2_	EtOH/60 °C, 2–240 min	75–95%	132
30	ZrOL_2_@SMNP	EtOH, rt, 5–50 min	73–97%	133
31	Nanosulfated zirconia	EtOH, reflux	75–90%	134
32	Cobalt NPs (Co-phen/C-800)	CsOH, H_2_O (0.75 eq.) toluene, 150 °C, 24 h	30–95%	137
33	Ni@Co_3_O_4_ nanocage	EtOH, 15–20 min 30 °C	97.5–100%	138
34	Cobalt nanoparticles on mesoporous SBA-15	EtOH reflux 90 min, air	88–95%	139
35	α-MoO_3_ nanobelts	EtOH, 60 °C	80–95%	142
36	Keplerate {Mo132} nanoball	EtOH, 60–75 °C, 5–90 min		143
37	Carbon-doped MoO_3_–TiO_2_ (CMT)	EtOH/H_2_O, 40 °C, 4–22 min	68–97%	144
38	*m*-MnO nanocrystals (1.5 mol%)	DMF, 130 °C	94%	147
39	MnFe_2_O_4_ NP (10%)	EtOH, 2.5 h, rt	70–91%	148
40	Ru complex supported on graphene oxide	HCOOH/NEt_3_, 70 °C, 2–24 h	75–92%	149
41	Ru/N–C, KOH	Toluene	21–88%	150
42	ZnO-KIT-6	MeOH, rt, 10–60 min	83–99%	151
43	Pd(ii)-Schiff base/SBA-15	H_2_O, 100 °C	94–99%	153
44	Na_2_PdP_2_O_7_	EtOH, rt	77–98%	154
45	Cs(CTA)_2_PW_12_O_40_	Solvent-free 10–90 min	62–99%	156
46	10 wt% TPA/MCM-41	EtOH, rt	65–99%	157

## Abbreviations

AMPA receptor: α-Amino-3-hydroxy-5-methyl-4-isoxazolepropionic acid antagonist
APTES: (3-Aminopropyl)tiethoxysilane
BET: Brunauer–Emmett–Teller
CNQX: 6-Cyano-7-nitroquinoxaline-2,3-dione
CNT: Carbon nanotube
CQS: Chloroquinoxaline sulfonamide
DCM: Dichloromethane
DMF: Dimethylformamide
DNA: Deoxyribonucleic acid
DRS: Differential reflectance spectroscopy
EDG: Electron-donating groups
EDX: Energy dispersive X-ray
EWG: Electron withdrawing groups
FTIR: Fourier transform infrared spectroscopy
GO: Graphene oxide
ICP: Inductively coupled plasma
MOF: Metal–organic frameworks
NBQX: 2,3-Dioxo-6-nitro-7-sulfamoyl-benzo[*f*]quinoxaline
nm: Nano meter
NP: Nano particle
OPD: *o*-Phenylenediamine
PBA: Bisphosphonic acid
POCl_3_: Phosphorus oxychloride
pXRD: Powder X-ray diffraction
rt: Room temperature
SBA-15: Santa Barbara Amorphous-15
SEM: Scanning electron microscopy
SiCN: Siliconcarbonitrite
SMNP: Starch coated maghemite nanoparticles
TEM: Transition electron microscopy
TGA: Thermogravimetric analysis
VSM: Vibrating sample magnetometer
XPS: X-ray photoelectron spectroscopy

## Ethical statement

No animals were used.

## Data availability

Data are contained in the manuscript. More data can be obtained from the corresponding author through request email.

## Author contribution

Dr Rangappa S. Keri: work design and written. Dr Dinesh Reddy: data collection and drawing structures. Dr Srinivasa: formalanalysis; validation; visualization. Dr Vinayak: correction and drawing.

## Conflicts of interest

The authors declared that they have no conflict of interest.

## Supplementary Material
